# Complex T-spherical fuzzy Dombi aggregation operators and their applications in multiple-criteria decision-making 

**DOI:** 10.1007/s40747-021-00446-2

**Published:** 2021-07-11

**Authors:** Faruk Karaaslan, Mohammed Allaw Dawood Dawood

**Affiliations:** Department of Mathematics, Faculty of Sciences, Çankırı Karatekin University, 18100 Çankırı, Turkey

**Keywords:** Complex fuzzy set, Spherical fuzzy set, Complex T-spherical fuzzy set, Dombi operators, Decision-making

## Abstract

Complex fuzzy (CF) sets (CFSs) have a significant role in modelling the problems involving two-dimensional information. Recently, the extensions of CFSs have gained the attention of researchers studying decision-making methods. The complex T-spherical fuzzy set (CTSFS) is an extension of the CFSs introduced in the last times. In this paper, we introduce the Dombi operations on CTSFSs. Based on Dombi operators, we define some aggregation operators, including complex T-spherical Dombi fuzzy weighted arithmetic averaging (CTSDFWAA) operator, complex T-spherical Dombi fuzzy weighted geometric averaging (CTSDFWGA) operator, complex T-spherical Dombi fuzzy ordered weighted arithmetic averaging (CTSDFOWAA) operator, complex T-spherical Dombi fuzzy ordered weighted geometric averaging (CTSDFOWGA) operator, and we obtain some of their properties. In addition, we develop a multi-criteria decision-making (MCDM) method under the CTSF environment and present an algorithm for the proposed method. To show the process of the proposed method, we present an example related to diagnosing the COVID-19. Besides this, we present a sensitivity analysis to reveal the advantages and restrictions of our method.

## Introduction

The fuzzy set (FS) theory was inaugurated by Zadeh [1] in 1965 to handle modelling of some problems containing uncertain data in real life. Since FS theory is a very useful tool for modelling uncertainty, it has many applications in the modelling and solving of the problems in many fields such as medical science, data mining and clustering. An FS is characterized by a membership function (MF) $\mu$ from a set of the objects or elements considered in the universe to the interval [0,1]. In an FS, if the membership degree (MD) of an element *x* is $\mu (x)$, then its non-membership degree (NMD) is $1-\mu (x)$, that is, in the FS, hesitation degree of an element is “0”. This is one of the limited aspects of FS in modelling real-life problems. To overcome these limitations, the intuitionistic FS (IFS) was suggested by Atanassov [2] as a generalization of FSs. An IFS is identified by two functions from a universal set to the interval [0,1] called membership function (MF) ($\mu$) and non-membership function (NMF) ($\nu$). The summation of images under these two functions of an element cannot exceed 1. Therefore, IFS is not an appropriate tool for modeling in the situation $\mu (x) + \nu (x) > 1$. To cope with this restriction, Yager [3, 4] introduced the concept of Pythagorean FS (PyFS) as an extension of IFS under condition $\mu ^2(x)+\nu ^2(x)\le 1$. However, in the situation $0.9^2+0.5^2=1.06>1$, a PyFS is not sufficient for modelling. To eliminate this type of limitation, Yager [5] put forward the concept of q-rung orthopair FS in which $\mu ^q(x)+\nu ^q(x)\le 1$. The neutral situation is not taken into account in the set theories we have mentioned so far, but this situation is important for the representation of human thinking. For this, Cuong [6, 7] defined the concept of the Picture FS (PFS). A PFS is a useful tool for expressing how much an object provides a feature or how much a person has shared an idea because a PFS does a modelling considering cases of yes, abstention, no, and rejection. A PFS is characterized by three values from interval [0,1] for each element x belonging to set containing considered elements, called MD ($\mu (x)$), abstinence degree (AD) or neutral degree ($\gamma (x)$) and, NMD ($\nu (x)$) with the condition $0\le \mu (x)+ \gamma (x)+ \nu (x)\le 1$. Despite the fact that PFS structure is a useful tool in many applications such as decision-making (DM) [8–14], similarity measure [15–19], correlation coefficient [20, 21], and clustering [22, 23], it is not sufficient in modelling of some problems because of constrain $0\le \mu (x)+ \gamma (x)+ \nu (x)\le 1$. Therefore, the notion of spherical FS (SFS), which is an extension of PFS, was initiated by Gungogdu and Kahraman [24, 25] and the applications of the SFS to decision-making was studied on. An SFS has the constrain $0\le \mu ^2(x)+\gamma ^2(x)+\nu ^2(x)\le 1$. Kahraman et al. [24] developed a DM method based on the TOPSIS method under the SF environment and presented an application of the developed method in the selection of hospital location. In an SFS, when MD, NeD and NMD of an element are taken as 0.6, 0.9 and 0.5, respectively, since $0.6^2+0.9^2+0.5^2=1.42>1$, condition $0\le \mu ^2(x)+\gamma ^2(x)+\nu ^2(x)\le 1$ is not satisfied. To model such situations, the T-spherical FS (T-SFS) was introduced by Mahmood et al. [26] as an extension of the SFS under condition X and some applications in medical diagnosis and DM problems under T-SF and SF environments were given by same researchers. After the works of Mahmood et al. [26], many researchers have studied applications of T-SFS and SFS. For example, Ullah et al. [27] proposed some novel similarity measures including cosine similarity measures, grey similarity measures, and set theoretic similarity measures for SFS and T-SFSs. Garg et al. [28] defined some new improved aggregation operators for T-SFSs and developed a DM approach to solve the multi-attribute DM (MADM) problems. Ullah et al. [29] introduced the some ordered weighted aggregation operators and hybrid aggregation operators of T-SFS and proposed an MADM method. Wu et al. [30] studied divergence measure of T-SFSs and gave the application in pattern recognition. Ullah et al. [31] defined the concept of interval-valued T-SFSs and their basic operations. They also described two aggregation operators for interval-valued T-SF values and developed an MADM method for problem including evaluating companies to be made an investment. Liu et al. [32] proposed some novel operational laws for T-SPFNs and combine power average operator and with Murihead mean operator. They also developed some new aggregation operators. Guleria and Bajaj [33] defined some aggregation operations of T-Spherical fuzzy soft sets. Quek et al. [34] presented some new operational laws for T-spherical fuzzy sets and obtain some of their properties. Then, based on these new operations, they have proposed two types of Einstein aggregation operators called the Einstein interactive averaging aggregation operators and the Einstein interactive geometric aggregation operators. They also put forward a MADM method based on the defined aggregation operators. Munir et al. [35] studied on Einstein hybrid aggregation operators under T-SF environment and establish an MADM by integrating the proposed aggregation operators. Ullah et al. [36] establish the correlation coefficient formula for T-SF values and presented an application in clustering. Also, T-spherical Fuzzy Hamacher Aggregation Operators were defined Ullah et al. [37]. Furthermore, they put forward an MADM method and gave the application of the method in a problem including evaluation of the performance of search and rescue robots. Garg et al. [38] introduced power aggregation operators for the T-spherical fuzzy sets (T-SFSs). Ju et al. [39] defined the T-SF interaction aggregation operators and based on these operators they developed TODIM method under T-SF environment. Chen et al. [40] studied on some generalized T-Spherical and group-Generalized fuzzy geometric aggregation operators with MADM method. Associated immediate probability (interactive) geometric aggregation operators of for T-spherical fuzzy sets were introduced by Munir et al. [41].

As mentioned above, FS models are important tools for modelling uncertain and incomplete data. But mentioned FS models do not suffice to express the periodic information or two-dimension phenomenon. To cope with this issue, the concept of complex FS (CFS) was put forward by Ramot et al. [42, 43]. The basic idea in the definition given by Ramot is to extend the range of membership from [0, 1] to the unit circle in the complex plane. A CFS is characterized by membership function $\mu =re^{i\omega }$ where *r* is called amplitude term and it takes values from the interval [0,1], and $\omega$ is called phase term (periodic term) and it lies in the interval $[0, 2\pi ]$. The phase term has a very important role in defining the CF model. This is what makes the CF sets superior and distinct from other FS models. In a CFS, the membership value of an element is specified based on one amplitude term and one phase term. With this aspect, CFS is not enough to model the nonmembership degree. To avoid this restriction, Alkouri and Salleh [44] introduced complex intuitionistic FS (CIFS). A CIFS is identified by MF ($\mu =re^{i\omega }$) and NMF ($\nu =ke^{i\eta }$) such that $0\le r+k\le 1$ and $0\le \omega +\eta \le 2\pi$. Rani and Garg [45] developed a DM approach based on distance measure between CIFSs. Also, some researchers studied on aggregation operator of CIFS and DM methods [46–51]. Additionally, Ullah et al. [52] introduced the complex PyFS (CPFS) which is characterized by MF $\mu =re^{i\omega }$, NeF $\mu =se^{i\theta }$, and NMF $\nu =ke^{i\eta }$ under the conditions $0\le r+s+k\le 1$ and $0\le \frac{\omega }{2\pi }+\frac{\theta }{2\pi }+\frac{\eta }{2\pi }\le 1$ as a generalization of CIFSs. Liu et al. [53] defined the complex q-ROFS (CqROFS) and studied on aggregation operator of them.

Akram et al. [54] presented some aggregation operators under CPF environment based on Hamacher operations and developed an MCDM method. Liu et al. [55] introduced CPF power averaging and CPF power geometric operators under CPFSs environment and constructed an MCDM method based on the proposed operators. Additionally, the complex SFS (CSFS) was defined by Akram et al. [56] as a generalization of CPFS. They also introduced some aggregation operation based on Dombi t-norm and t-conorm. Ali et al. [57] defined the concept of complex T-spherical FS (CTSFS) and their aggregation operators. They also proposed an MADM method in CTSFSs. Akram et al. [58] defined some aggregation operators of CSFSs and developed an MCGDM method called CSF-VIKOR. Nasir et al. [59] introduced the notion of CTSF relations and presented some applications related to the economy and international trade.

Since aggregation operators (AOs) convert the whole data into a single value, AOs have a vital importance in DM problems. Dombi [60] designated Dombi operators with flexible operational variables. In solving the DM problems, many researchers used Dombi operations of IFS [61], Pythagorean fuzzy [62–64], PF [66], bipolar fuzzy [65], spherical fuzzy [67], complex Pythagorean [68], and CSF [69].

As seen above, the studies on the theoretical aspects of SFSs, T-SFSs, CSFSs and CTSFSs and their applications in decision-making based on aggregation operators have increased rapidly. The following points motivate us to present this paper:

- CFS and its generalizations have a very important role in decision-making problems containing two-dimensional information in real life. A TSFS comprehends a large amount of information as a generalization of the SFSs. However, it does not suffice in modelling an issue involving two-dimensional data. With this aspect, CTSFS has vital importance. A CTSFS is the generalization of theories like CFS, CIFS, CPFS, CPyFS and CSFS. Until now, there exists only one work [57] related to aggregation operators of CTSFS in the literature. Therefore, by considering the advantages of the Dombi operators, we develop some new aggregation operators based on Dombi t-norm and t-conorm to use in modelling a problem involving two-dimensional data.
- Set-theoretical operators are an important tool for modelling some problems, in the literature, there is not any study related to set-theoretical operations of CTSFS. To fill this gap in the literature, we define the set-theoretical operations of CTSFSs.
- In literature, there is only one study related to score and accuracy functions of CTSFNs and these functions have some drawbacks, we pointed out these drawbacks and define novel score and accuracy functions free from specified drawbacks.
- We see that works related to aggregation operators of SFS, TSFS and CTSFSs are based on the hypothetical data in general. In this study, one of our aims is to develop a decision-making method by considering the advantages of the Dombi operators and presenting an application including real data that aims to diagnose COVID-19 patients.

This article is organized as follows: the next section recalls the required definitions in the following sections as SFS, TSFS, CTSFSs and Dombi operations. Also, new score and accuracy functions and set-theoretical operation are defined. The subsequent section defines Dombi operations of complex T-spherical fuzzy numbers and provides their examples, and related operations of these operators are obtained for introduced aggregation operators. Then the MCDM method and its application are presented. Before the final section, sensitivity analyses and discussion related to obtained results from the application of the proposed method are given. The final section mentions the conclusions and planned studies.

## Preliminaries

This section reminds the definitions of CFS, CIFS, CPyFS, CPFS, SFS, T-SFS and CTSFSs.

### Definition 1

[42] Let ${\mathfrak {X}}$ be a nonempty set. A complex fuzzy set (CFS) $\mathfrak {I}$ is defined as

$$\begin{aligned} {\mathcal {F}}=\{({\mathfrak {x}},{\tilde{\alpha }}_{{\mathcal {F}}}({\mathfrak {x}})):{\mathfrak {x}}\in {\mathfrak {X}}\}, \end{aligned}$$

where ${\tilde{\alpha }}_{{\mathcal {F}}}({\mathfrak {x}})$ is called membership functions of CFS ${\mathcal {F}}$ and receive all lying within the unit circle in the complex plane. Thus, it can be expressed as ${\tilde{\alpha }}_{{\mathcal {F}}}({\mathfrak {x}})=\alpha _{{\mathcal {F}}}({\mathfrak {x}})e^{i2\pi \varpi _{\alpha _{{\mathcal {F}}}}({\mathfrak {x}})}$, and it denotes a complex-valued grade of membership of ${\mathfrak {x}}\in {\mathfrak {X}}$ to (CFS) $\mathfrak {I}$. Here $i=\sqrt{-1}$ and for all ${\mathfrak {x}}\in {\mathfrak {X}}$, $0\le \alpha _{{\mathcal {F}}}({\mathfrak {x}}) \le 1$, and $0\le \varpi _{\alpha _{{\mathcal {F}}}}({\mathfrak {x}})\le 1.$

### Definition 2

[44] Let ${\mathfrak {X}}$ be a nonempty set. A complex intuitionistic fuzzy set (CIFS) ${\mathcal {I}}$ is defined as:

$$\begin{aligned} {\mathcal {I}}=\{({\mathfrak {x}},{\tilde{\alpha }}_{{\mathcal {I}}}({\mathfrak {x}}),{\tilde{\gamma }}_{{\mathcal {I}}}({\mathfrak {x}})):{\mathfrak {x}}\in {\mathfrak {X}}\}, \end{aligned}$$

where ${\tilde{\alpha }}_{{\mathcal {I}}}({\mathfrak {x}})$ and ${\tilde{\gamma }}_{{\mathcal {I}}}({\mathfrak {x}})$ are called membership function and non-membership function of CIFS ${\mathcal {I}}$, respectively. They receive all lying within the unit circle in the complex plane. Hence, they can be expressed as ${\tilde{\alpha }}_{{\mathcal {I}}}({\mathfrak {x}})= \alpha _{{\mathcal {I}}}({\mathfrak {x}})e^{i2\pi \varpi _{\alpha _{{\mathcal {I}}}}({\mathfrak {x}})}$, and ${\tilde{\gamma }}_{{\mathcal {I}}}({\mathfrak {x}})=\gamma _{{\mathcal {I}}}({\mathfrak {x}})e^{i2\pi \varpi _{\gamma _{{\mathcal {I}}}}({\mathfrak {x}})},$ where they denote the complex-valued grades of membership and non-membership of ${\mathfrak {x}}\in {\mathfrak {X}}$ to CIFS ${\mathcal {I}}$, respectively. Here $i=\sqrt{-1}$, for all ${\mathfrak {x}}\in {\mathfrak {X}}$, $0\le \alpha _{{\mathcal {I}}}({\mathfrak {x}})+\gamma _{{\mathcal {I}}}({\mathfrak {x}}) \le 1$, and $0\le \varpi _{\alpha _{{\mathcal {I}}}}({\mathfrak {x}})+\varpi _{\gamma _{{\mathcal {I}}}}({\mathfrak {x}})\le 1.$

### Definition 3

[52] Let ${\mathfrak {X}}$ be a nonempty set. A complex pythagorean fuzzy set (CPyFS) ${\mathfrak {P}}$ is defined as

$$\begin{aligned} {\mathfrak {P}} =\{({\mathfrak {x}},{\tilde{\alpha }}_{{\mathfrak {P}} },{\tilde{\gamma }}_{{\mathfrak {P}}}({\mathfrak {x}})):{\mathfrak {x}}\in {\mathfrak {X}}\}, \end{aligned}$$

where ${\tilde{\alpha }}_{{\mathfrak {P}}}({\mathfrak {x}})$ and ${\tilde{\gamma }}_{{\mathfrak {P}} }({\mathfrak {x}})$ are called complex-valued membership function and non-membership function of CPyFS ${\mathfrak {P}}$, respectively. They receive all lying within the unit circle in the complex plane. Thus, they can be expressed as ${\tilde{\alpha }}_{{\mathfrak {P}}}({\mathfrak {x}})=\alpha _{{\mathfrak {P}} }({\mathfrak {x}})e^{i2\pi \varpi _{\alpha _{{\mathfrak {P}}}}({\mathfrak {x}})}$, and ${\tilde{\gamma }}_{{\mathfrak {P}} }({\mathfrak {x}})=\gamma _{{\mathfrak {P}}}({\mathfrak {x}})e^{i2\pi \varpi _{\gamma _{{\mathfrak {P}} }}({\mathfrak {x}})},$ where they denote the complex-valued grades of membership and non-membership of ${\mathfrak {x}}\in {\mathfrak {X}}$ to CPyFS ${\mathfrak {P}}$, respectively. Here $i=\sqrt{-1}$ and for all ${\mathfrak {x}}\in {\mathfrak {X}}$, $0\le \alpha _{{\mathcal {I}}}^2({\mathfrak {x}})+\gamma _{{\mathcal {I}}}^2({\mathfrak {x}}) \le 1$, and $0\le \varpi _{\alpha _{{\mathfrak {P}} }}^2({\mathfrak {x}})+\varpi _{\gamma _{{\mathfrak {P}}}}^2({\mathfrak {x}})\le 1$ .

### Definition 4

[54] Let ${\mathfrak {X}}$ be a nonempty set. A complex picture fuzzy set (CPFS) ${\mathcal {P}}$ is defined as

$$\begin{aligned} {\mathcal {P}}=\{({\mathfrak {x}},{\tilde{\alpha }}_{{\mathcal {P}}}({\mathfrak {x}}),{\tilde{\beta }}_{{\mathcal {P}}}({\mathfrak {x}}), {\tilde{\gamma }}_{{\mathcal {P}}}({\mathfrak {x}})):{\mathfrak {x}}\in {\mathfrak {X}}\}, \end{aligned}$$

where $\alpha _{{\mathcal {P}}}({\mathfrak {x}}), \beta _{{\mathcal {P}}}({\mathfrak {x}})$, and $\gamma _{{\mathcal {P}}}({\mathfrak {x}})$ are called membership, neutral membership, and non-membership function of the CPFS ${\mathcal {P}}$, respectively. They receive all lying within the unit circle in the complex plane. Thus, they can be expressed as ${\tilde{\alpha }}_{{\mathcal {P}}}({\mathfrak {x}})=\alpha _{{\mathcal {P}}}({\mathfrak {x}})e^{i2\pi \varpi _{\alpha _{{\mathcal {P}}}}({\mathfrak {x}})}$, ${\tilde{\beta }}_{{\mathcal {P}}}({\mathfrak {x}})=\beta _{{\mathcal {P}}}({\mathfrak {x}})e^{i2\pi \varpi _{\beta _{{\mathcal {P}}}}({\mathfrak {x}})}$, and ${\tilde{\gamma }}_{{\mathcal {P}}}({\mathfrak {x}})=\gamma _{{\mathcal {P}}}({\mathfrak {x}})e^{i2\pi \varpi _{\gamma _{{\mathcal {P}}}}({\mathfrak {x}})},$ where they denote the complex-valued grades of membership and non-membership of ${\mathfrak {x}}\in {\mathfrak {X}}$ to CIFS ${\mathcal {P}}$, respectively. Here $i=\sqrt{-1}$, and for all ${\mathfrak {x}}\in {\mathfrak {X}}$, $0\le \alpha _{{\mathcal {P}}}({\mathfrak {x}})+\beta _{{\mathcal {P}}}({\mathfrak {x}})+\gamma _{{\mathcal {P}}}({\mathfrak {x}}) \le 1$, and $0\le \varpi _{\alpha _{{\mathcal {P}}}}({\mathfrak {x}})+\varpi _{\beta _{{\mathcal {P}}}}({\mathfrak {x}})+\varpi _{\gamma _{{\mathcal {P}}}}({\mathfrak {x}})\le 1.$

### Definition 5

[24, 25] Let ${\mathfrak {X}}$ be a non-empty set. A spherical fuzzy set (SFS) ${\mathcal {A}}$ is defined over ${\mathfrak {X}}$ as follows:

$$\begin{aligned} {\mathcal {A}}= & {} \Big \{\Big ({\mathfrak {x}},\alpha _{{\mathcal {A}}}({\mathfrak {x}}),\\&\beta _{{\mathcal {A}}}({\mathfrak {x}}),\gamma _{{\mathcal {A}}}({\mathfrak {x}})\Big ):0\\\le & {} \alpha _{{\mathcal {A}}}^2({\mathfrak {x}})+\beta _{{\mathcal {A}}}^2({\mathfrak {x}})\\&+\gamma _{{\mathcal {A}}}^2({\mathfrak {x}})\le 1, {\mathfrak {x}}\in {\mathfrak {X}}\Big \}. \end{aligned}$$

Here the function $\alpha _{{\mathcal {A}}}:{\mathfrak {X}} \rightarrow [0,1]$ expresses MF, $\beta _{{\mathcal {A}}}:{\mathfrak {X}}\rightarrow [0,1]$ expresses NeMF, and $\gamma _{{\mathcal {A}}}:X \rightarrow [0,1]$ expresses NMF of the SFS ${\mathcal {A}}$.

The concept of T-spherical fuzzy set was introduced by Mahmood et al. [26] as a generalization of the SFSs, as follows:

### Definition 6

[26] Let ${\mathfrak {X}}$ be a nonempty set. A T-spherical fuzzy (TSF) set (TSFS) is defined over ${\mathfrak {X}}$ as follows:

$$\begin{aligned} {\mathcal {T}}= & {} \Big \{\Big ({\mathfrak {x}},\alpha _{{\mathcal {T}}}({\mathfrak {x}}),\beta _{{\mathcal {T}}}({\mathfrak {x}}),\\&\gamma _{{\mathcal {T}}}({\mathfrak {x}})\Big ):0\le \\&\alpha _{{\mathcal {T}}}^q({\mathfrak {x}})+\beta _{{\mathcal {T}}}^q({\mathfrak {x}})\\&+\gamma _{{\mathcal {T}}}^q({\mathfrak {x}})\le 1, {\mathfrak {x}}\in {\mathfrak {X}}\Big \}. \end{aligned}$$

Here the function $\alpha _{{\mathcal {A}}}:{\mathfrak {X}} \rightarrow [0,1]$, $\beta _{{\mathcal {T}}}:{\mathfrak {X}}\rightarrow [0,1]$, and $\gamma _{{\mathcal {A}}}:{\mathfrak {X}} \rightarrow [0,1]$ express MF, NeMF, and NMF of the TSFS ${\mathcal {T}}$, respectively.

### Definition 7

[57] Let ${\mathfrak {X}}$ be an initial universe different from empty set. A complex T-spherical fuzzy (CTSF) set (CTSFS) is defined as follows:

$$\begin{aligned} {\digamma }=\{({\mathfrak {x}}, \alpha _\digamma ({\mathfrak {x}}), \beta _\digamma ({\mathfrak {x}}), \gamma _\digamma ({\mathfrak {x}})): {\mathfrak {x}}\in {\mathfrak {X}}\}. \end{aligned}$$

Here $\alpha _\digamma ({\mathfrak {x}})=\alpha _\digamma ({\mathfrak {x}})e^{i2\pi \varpi _{\beta _\digamma ({\mathfrak {x}})}}$, $\beta _\digamma ({\mathfrak {x}})=\beta _\digamma ({\mathfrak {x}})e^{i2\pi \varpi _{\beta _\digamma ({\mathfrak {x}})}}$, and $\gamma _\digamma ({\mathfrak {x}})=\gamma _\digamma ({\mathfrak {x}})e^{i2\pi \varpi _{\gamma _\digamma ({\mathfrak {x}})}}$ denote the membership grades of truth, abstinence, and falsity such that $0\le \alpha ^q_\digamma ({\mathfrak {x}})+\beta ^q_\digamma ({\mathfrak {x}})+\gamma ^q_\digamma ({\mathfrak {x}})\le 1$ and $0\le \varpi ^q_{\alpha _\digamma ({\mathfrak {x}})}$ $+\varpi ^q_{\beta _\digamma ({\mathfrak {x}})}+\varpi ^q_{\gamma _\digamma ({\mathfrak {x}})}\le 1.$ Furthermore,

${\mathfrak {H}}_\digamma ({\mathfrak {x}})=\root q \of {1-\alpha _\digamma ({\mathfrak {x}})^q-\beta _\digamma ({\mathfrak {x}})^q-\alpha _\digamma ({\mathfrak {x}})^q} e^{\root q \of {(1-\varpi _{\alpha _\digamma ({\mathfrak {x}})}^q-\varpi _{\beta _\digamma ({\mathfrak {x}})}^q -\varpi _{\gamma _\digamma ({\mathfrak {x}})}^q)}}$ expresses the complex hesitancy grade of ${\mathfrak {x}}.$

For convenience, $\digamma =(\alpha _ke^{i2\pi \varpi _{\alpha _k}},\beta _ke^{i2\pi \varpi _{\beta _k}},\gamma _ke^{i2\pi \varpi _{\gamma _k}})$ is called complex T-spherical fuzzy number (CTSFN).

Ali et al. [57] defined the score functions for the CTSFNs by taking absolute value of formula given the following definitions.

$$\begin{aligned} \Omega (\digamma )= \frac{1}{2}|(\alpha ^{q}- \beta ^{q}- \gamma ^{q})+(\varpi _{\alpha ^q}- \varpi _{\beta ^q}-\varpi _{\gamma ^q})|\\ \mho (\digamma )= \frac{1}{2}|(\alpha ^{q}+ \beta ^{q}+ \gamma ^{q})+(\varpi _{\alpha ^q}+ \varpi _{\beta ^q}+\varpi _{\gamma ^q})|. \end{aligned}$$

When we consider the CTSFNs $(1e^{i2\pi 1},0e^{i2\pi 0},0e^{i2\pi 0})$ and $(0e^{i2\pi 1},0e^{i2\pi 0},1e^{i2\pi 1})$, their score values are 1. So, we need to use the accuracy function, but their accuracy values are 1. This is a weak aspect of the proposed score and accuracy functions. Therefore, we define the following score and accuracy functions.

### Definition 8

Let $\digamma =(\alpha e^{i2\pi \varpi _{\alpha }},\beta e^{i2\pi \varpi _{\beta }},\gamma e^{i2\pi \varpi _{\gamma }})$ is a *CTSFN*. The score function $\Omega (\digamma )$ and accuracy function $\mho (\digamma )$ of $\digamma$ are formulated as follows:

1 $$\begin{aligned} \Omega (\digamma )= & {} \frac{1}{4}\Big (2+(\alpha ^{q}- \beta ^{q}- \gamma ^{q})+(\varpi _{\alpha ^q}- \varpi _{\beta ^q}-\varpi _{\gamma ^q})\Big ) \nonumber \\\end{aligned}$$

2 $$\begin{aligned} \mho (\digamma )= & {} \frac{1}{4}\Big (2+(\alpha ^{q}+ \beta ^{q}+ \gamma ^{q})+(\varpi _{\alpha ^q}+ \varpi _{\beta ^q}+\varpi _{\gamma ^q})\Big ).\nonumber \\ \end{aligned}$$

### Definition 9

Let $\digamma _{1}=(\alpha _1e^{i2\pi \varpi _{\alpha _1}},\beta _1e^{i2\pi \varpi _{\beta _1}},\gamma _1e^{i2\pi \varpi _{\gamma _1}})$ and $\digamma _{2}=(\alpha _2e^{i2\pi \varpi _{\alpha _2}},\beta _2e^{i2\pi \varpi _{\beta _2}},\gamma _2e^{i2\pi \varpi _{\gamma _2}})$ are two CTSFNs. For the comparison of $\digamma _{1}$ and $\digamma _{2}$,

- $\digamma _{1} \succeq \digamma _{2}$ ($\digamma _{1}$ is superior to $\digamma _{2}$) if $\Omega (\digamma _{1})> \Omega (\digamma _{2})$;
- if $$\Omega (\digamma _{1}) = \Omega (\digamma _{2})$$, then
  - $\digamma _{1} \succeq \digamma _{2}$ ($\digamma _{1}$ is superior to $\digamma _{2}$) if $\mho (\digamma _{1})> \mho (\digamma _{2})$;
  - $\digamma _{1} \sim \digamma _{2}$ ($\digamma _{1}$ is equivalent to $\digamma _{2}$) if $\mho (\digamma _{1}) = \mho (\digamma _{2})$.

### Definition 10

Let $\digamma _{1}=\Big \{\Big ({\mathfrak {x}},\alpha _1({\mathfrak {x}})e^{i2\pi \varpi _{\alpha _1}({\mathfrak {x}})}, \beta _1({\mathfrak {x}})e^{i2\pi \varpi _{\beta _1}({\mathfrak {x}})},\gamma _1({\mathfrak {x}})e^{i2\pi \varpi _{\gamma _1}({\mathfrak {x}})}\Big ):{\mathfrak {x}}\in {\mathfrak {X}}\Big \}$ and $\digamma _2=\Big \{\Big ({\mathfrak {x}},\alpha _2({\mathfrak {x}})e^{i2\pi \varpi _{{\alpha _2}({\mathfrak {x}})}},\beta _2({\mathfrak {x}}) e^{i2\pi \varpi _{{\beta _2}({\mathfrak {x}})}},\gamma _2({\mathfrak {x}})e^{i2\pi \varpi _{{\gamma _2}({\mathfrak {x}})}}\Big ): {\mathfrak {x}}\in {\mathfrak {X}}\Big \}$ be any two CTSFSs. Then

1. $\digamma _{1} \preceq \digamma _{2}$ iff $\alpha _1({\mathfrak {x}}) \le \alpha _2({\mathfrak {x}})$,   $\beta _1({\mathfrak {x}})\le \beta _2({\mathfrak {x}})$,  $\gamma _1({\mathfrak {x}}) \ge \gamma _2({\mathfrak {x}})$ and $\varpi _{{\alpha _1}({\mathfrak {x}})}\le \varpi _{{\alpha _2}({\mathfrak {x}})}$,$\varpi _{{\beta _1}({\mathfrak {x}}})\le \varpi _{{\beta _2}({\mathfrak {x}})}$,$\varpi _{{\gamma _1}({\mathfrak {x}})}\ge \varpi _{{\gamma _2}({\mathfrak {x}})}$, for all ${\mathfrak {x}}\in {\mathfrak {X}}.$
2. $$(\digamma _{1})^{c}=\{({\mathfrak {x}},\gamma _1({\mathfrak {x}})e^{i2\pi \varpi _{{\gamma _1}({\mathfrak {x}})}},\beta _1({\mathfrak {x}}) e^{i2\pi \varpi _{{\beta _1}({\mathfrak {x}})}},\alpha _1({\mathfrak {x}})e^{i2\pi \varpi _{{\alpha _1}({\mathfrak {x}})}}): {\mathfrak {x}}\in {\mathfrak {X}}\}.$$
3. $$\begin{aligned} \digamma _{1} \cup \digamma _{2}= & {} \Big \{\Big ({\mathfrak {x}},\mathrm{max}\{\alpha _1({\mathfrak {x}}),\alpha _2({\mathfrak {x}})\}e^{i2\pi \mathrm{max}\{\varpi _{{\alpha _1}({\mathfrak {x}})},\varpi _{{\alpha _2}({\mathfrak {x}})}\}}, \\&\mathrm{min}\{\beta _1({\mathfrak {x}}), \beta _2({\mathfrak {x}})\}e^{i2\pi \mathrm{min}\{\varpi _{{\beta _1}({\mathfrak {x}})},\varpi _{{\beta _2}({\mathfrak {x}})}\}},\\&\mathrm{min}\{ \gamma _1({\mathfrak {x}}),\gamma _2({\mathfrak {x}})\}e^{i2\pi \mathrm{min}\{\varpi _{{\gamma _1}({\mathfrak {x}})},\varpi _{{\gamma _2}({\mathfrak {x}})}\}}\Big ):\\&{\mathfrak {x}}\in {\mathfrak {X}}\Big \}. \end{aligned}$$
4. $$\begin{aligned} \digamma _{1} \cap \digamma _{2}= & {} \Big \{\Big ({\mathfrak {x}},\mathrm{min}\{\alpha _1({\mathfrak {x}}),\alpha _2({\mathfrak {x}})\}e^{i2\pi \mathrm{min}\{\varpi _{{\alpha _1}({\mathfrak {x}})},\varpi _{{\alpha _2}({\mathfrak {x}})}\}}, \\&\mathrm{min}\{\beta _1({\mathfrak {x}}), \beta _2({\mathfrak {x}})\}e^{i2\pi \mathrm{min}\{\varpi _{{\beta _1}({\mathfrak {x}})},\varpi _{{\beta _2}({\mathfrak {x}})}\}},\\&\mathrm{max}\{ \gamma _1({\mathfrak {x}}),\gamma _2({\mathfrak {x}})\}e^{i2\pi \mathrm{max}\{\varpi _{{\gamma _1}({\mathfrak {x}})},\varpi _{{\gamma _2}({\mathfrak {x}})}\}}\Big ):\\&{\mathfrak {x}}\in {\mathfrak {X}}\Big \}. \end{aligned}$$

### Example 1

Let us consider CTSFSs $\digamma _{1}$ and $\digamma _{2}$ over universal set ${\mathfrak {X}}=\{{\mathfrak {x}}_{1},{\mathfrak {x}}_{2},{\mathfrak {x}}_{3}\}$ given as follows:

$$\begin{aligned} \digamma _{1}= & {} \Big \{\Big ({\mathfrak {x}}_{1},0.8e^{i2\pi 0.81},0.5e^{i.2\pi 0.52},0.9e^{i2\pi 0.93}\Big ),\\&\Big ({\mathfrak {x}}_{2},0.7e^{i2\pi 0.72},0.7e^{i2\pi 0.73},0.9e^{i2\pi 0.92}\Big ),\\&\Big ({\mathfrak {x}}_{3},0.8e^{i2\pi 0.82},0.7e^{i2\pi 0.71},0.9e^{i2\pi 0.91}\Big )\Big \} \end{aligned}$$

and

$$\begin{aligned} \digamma _{2}= & {} \Big \{\Big ({\mathfrak {x}}_{1},0.9e^{i2\pi 0.91},0.6e^{i2\pi 0.62},0.8e^{i2\pi 0.83}\Big ),\\&\Big ({\mathfrak {x}}_{2},0.8e^{i2\pi 0.81},0.8e^{i2\pi 0.82},0.7e^{i2\pi 0.72}\Big ),\\&\Big ({\mathfrak {x}}_{3},0.9e^{i2\pi 0.91},0.8e^{i2\pi 0.81},0.7e^{i.2\pi 0.71}\Big )\Big \}. \\ \end{aligned}$$

Then it is clear that $\digamma _{1}\subseteq \digamma _{2}$. Also,

$$\begin{aligned} (\digamma _{1})^{c}= & {} \Big \{\Big (x_{1},0.9e^{i2\pi 0.93},0.5e^{i2\pi 0.52},0.8e^{i2\pi 0.81}\Big ),\\&\Big ({\mathfrak {x}}_{2},0.9e^{i2\pi 0.92},0.7e^{i2\pi 0.73},0.7e^{i2\pi 0.72}\Big ),\\&\Big ({\mathfrak {x}}_{3},0.9e^{i2\pi 0.91},0.7e^{i2\pi 0.71},0.8e^{i2\pi 0.82}\Big )\Big \},\\ \digamma _{1}\cup \digamma _{2}= & {} \Big \{\Big ({\mathfrak {x}}_{1},0.9e^{i2\pi 0.91},0.5e^{i2\pi 0.52},\\&0.8e^{i2\pi 0.81}\Big ),\\&\Big ({\mathfrak {x}}_{2},0.8e^{i2\pi 0.81},0.7e^{i2\pi 0.73},0.7e^{i2\pi 0.72}\Big ),\\&\Big ({\mathfrak {x}}_{3},0.9e^{i.2\pi 0.91},0.7e^{i.2\pi 0.71},0.7e^{i.2\pi 0.71}\Big )\Big \}, \end{aligned}$$

and

$$\begin{aligned} \digamma _{1}\cap \digamma _{2}= & {} \Big \{\Big ({\mathfrak {x}}_{1},0.8e^{i2\pi 0.81},0.5e^{i2\pi 0.52},0.9e^{i2\pi 0.91}\Big ),\\&\Big ({\mathfrak {x}}_{2},0.7e^{i2\pi 0.72},0.7e^{i2\pi 0.73},0.7e^{i2\pi 0.71}\Big ),\\&\Big ({\mathfrak {x}}_{3},0.8e^{i2\pi 0.82},0.9e^{i2\pi 0.91},0.9e^{i2\pi 0.91}\Big )\Big \}. \end{aligned}$$

## Dombi operations of complex T-spherical fuzzy numbers

In this section, we remind the definitions of Dombi t-norm (TN) and t-conorm (TCN) defined in [60] and we define the arithmetic operations of CTSFNs using Dombi TN and TCN.

### Definition 11

[60] Let *f* and *g* be two real numbers. Then Dombi TN and Dombi TCN are defined by

$$\begin{aligned} \dfrac{1}{1+\bigg (\big ( \dfrac{1-g}{g}\big )^{\eta }+\big ( \dfrac{1-h}{h}\big )^{\eta }\bigg ) ^{\dfrac{1}{\eta }}}, ~~\eta > 0. \end{aligned}$$

Dombi t-conorm [60] is given by:

$$\begin{aligned} 1- \dfrac{1}{1+\bigg (\big (\dfrac{g}{1-g}\big )^{ \eta }+\big (\dfrac{h}{1-h}\big )^{ \eta }\bigg )^{\dfrac{1}{\eta }}}, ~~\eta >0, \end{aligned}$$

respectively.

### Definition 12

Let ${\mathfrak {X}}$ be a universe and $\digamma _1=\Big (\alpha _1e^{i2\pi \varpi _{\alpha _1}},\beta _1e^{i2\pi \varpi _{\beta _1}},\gamma _1e^{i2\pi \varpi _{\gamma _1}}\Big )$and $\digamma _2=\Big (\alpha _2e^{i2\pi \varpi _{\alpha _2}},\beta _2e^{i2\pi \varpi _{\beta _2}},\gamma _2e^{i2\pi \varpi _{\gamma _2}}\Big )$ are two CTSFNs on ${\mathfrak {X}}$. Then some Dombi operations between $\digamma _{1}$ and $\digamma _{2}$ are given as follows:

1. $$\digamma _1\bigoplus \digamma _2=\left( \begin{array}{c} \left( \begin{array}{c} \root q \of {1-\frac{1}{1+\big (\big (\frac{(\alpha _1)^q}{1-(\alpha _1)^q}\big )^{\eta }+\big (\frac{(\alpha _2)^q}{1-(\alpha _2)^q}\big )^{\eta }\big )^{\frac{1}{\eta }}}} e^{{i2\pi \root q \of {1-\frac{1}{1+\big (\frac{(\varpi _{\alpha _{1}})^q}{1-(\varpi _{\alpha _{1}})^q}\big )^{\eta }+ (\big (\frac{(\varpi _{\alpha _{2}})^q}{1-(\varpi _{\alpha _{2}})^q}\big )^{\eta }\big )^{\frac{1}{\eta }}}}}} \end{array} \right) ,\\ \left( \begin{array}{c} \root q \of {\frac{1}{1+\big (\big (\frac{1-(\beta _1)^q}{(\beta _1)^q}\big )^{\eta }+\big (\frac{1-(\beta _2)^q}{(\beta _2)^q}\big )^{\eta }\big )^{\frac{1}{\eta }}}} e^{i2\pi \root q \of {\frac{1}{1+\big (\frac{1-(\varpi _{\beta _{1}})^q}{(\varpi _{\beta _{1}})^q}\big )^{\eta }+ (\big (\frac{1-(\varpi _{\beta _{2}})^q}{(\varpi _{\beta _{2}})^q}\big )^{\eta }\big )^{\frac{1}{\eta }}}}} \end{array} \right) ,\\ \left( \begin{array}{c} \root q \of {\frac{1}{1+\big (\big (\frac{1-(\gamma _1)^q}{(\gamma _1)^q}\big )^{\eta }+\big (\frac{1-(\gamma _2)^q}{(\gamma _2)^q}\big )^{\eta }\big )^{\frac{1}{\eta }}}} e^{i2\pi \root q \of {\frac{1}{1+\big (\frac{1-(\varpi _{\gamma _{1}})^q}{(\varpi _{\gamma _{1}})^q}\big )^{\eta }+ (\big (\frac{1-(\varpi _{\gamma _{2}})^q}{(\varpi _{\gamma _{2}})^q}\big )^{\eta }\big )^{\frac{1}{\eta }}}}} \end{array} \right) , \end{array} \right) .$$
2. $$\digamma _1\bigotimes \digamma _2=\left( \begin{array}{c} \left( \begin{array}{c} \root q \of {\frac{1}{1+\big (\big (\frac{1-(\alpha _1)^q}{(\alpha _1)^q}\big )^{\eta }+\big (\frac{1-(\alpha _2)^q}{(\alpha _2)^q}\big )^{\eta }\big )^{\frac{1}{\eta }}}} e^{i2\pi \root q \of {\frac{1}{1+\big (\frac{1-(\varpi _{\alpha _{1}})^q}{(\varpi _{\alpha _{1}})^q}\big )^{\eta }+ (\big (\frac{1-(\varpi _{\alpha _{2}})^q}{(\varpi _{\alpha _{2}})^q}\big )^{\eta }\big )^{\frac{1}{\eta }}}}} \end{array} \right) ,\\ \left( \begin{array}{c} \root q \of {\frac{1}{1+\big (\big (\frac{1-(\beta _1)^q}{(\beta _1)^q}\big )^{\eta }+\big (\frac{1-(\beta _2)^q}{(\beta _2)^q}\big )^{\eta }\big )^{\frac{1}{\eta }}}} e^{i2\pi \root q \of {\frac{1}{1+\big (\frac{1-(\varpi _{\beta _{1}})^q}{(\varpi _{\beta _{1}})^q}\big )^{\eta }+ (\big (\frac{1-(\varpi _{\beta _{2}})^q}{(\varpi _{\beta _{2}})^q}\big )^{\eta }\big )^{\frac{1}{\eta }}}}} \end{array} \right) ,\\ \left( \begin{array}{c} \root q \of {1-\frac{1}{1+\big (\big (\frac{(\gamma _1)^q}{1-(\gamma _1)^q}\big )^{\eta }+\big (\frac{(\gamma _2)^q}{1-(\gamma _2)^q}\big )^{\eta }\big )^{\frac{1}{\eta }}}} e^{i2\pi \root q \of {1-\frac{1}{1+\big (\frac{(\varpi _{\gamma _{1}})^q}{1-(\varpi _{\gamma _{1}})^q}\big )^{\eta }+ (\big (\frac{(\varpi _{\gamma _{2}})^q}{1-(\varpi _{\gamma _{2}})^q}\big )^{\eta }\big )^{\frac{1}{\eta }}}}} \end{array} \right) , \end{array} \right) .$$
3. $$\tau \digamma =\left( \begin{array}{c} \left( \begin{array}{c} \root q \of {1-\frac{1}{1+\big (\tau \big (\frac{(\alpha _1)^q}{1-(\alpha _1)^q}\big )^{\eta }\big )^{\frac{1}{\eta }}}} e^{i2\pi \root q \of {1-\frac{1}{1+\big (\tau \big (\frac{(\varpi _{\alpha _{1}})^q}{1-(\varpi _{\alpha _{1}})^q}\big )^{\eta }\big )^{\frac{1}{\eta }}}}} \end{array} \right) ,\\ \left( \begin{array}{c} \root q \of {\frac{1}{1+\big (\tau \big (\frac{1-(\beta _1)^q}{(\beta _1)^q}\big )^{\eta }\big )^{\frac{1}{\eta }}}} e^{(i2\pi (\root q \of {\frac{1}{1+\big (\tau \big (\frac{1-(\varpi _{\beta _{1}})^q}{(\varpi _{\beta _{1}})^q}\big )^{\eta }\big )^{\frac{1}{\eta }}}}} \end{array} \right) ,\\ \left( \begin{array}{c} \root q \of {\frac{1}{1+\big (\tau \big (\frac{1-(\gamma _1)^q}{(\gamma _1)^q}\big )^{\eta }\big )^{\frac{1}{\eta }}}} e^{i2\pi \root q \of {\frac{1}{1+\big (\tau \big (\frac{1-(\varpi _{\gamma _{1}})^q}{(\varpi _{\gamma _{1}})^q}\big )^{\eta }\big )^{\frac{1}{\eta }}}}} \end{array} \right) \end{array}\right) ;\tau \ge 0.$$
4. $$\digamma ^{\tau }=\left( \begin{array}{c} \left( \begin{array}{c} \root q \of {\frac{1}{1+\big (\tau \big (\frac{1-(\alpha _1)^q}{(\alpha _1)^q}\big )^{\eta }\big )^{\frac{1}{\eta }}}} e^{\i 2\pi \root q \of {\frac{1}{1+\big (\tau \big (\frac{1-(\varpi _{\alpha _{1}})^q}{(\varpi _{\alpha _{1}})^q}\big )^{\eta }\big )^{\frac{1}{\eta }}}}} \end{array} \right) ,\\ \left( \begin{array}{c} \root q \of {\frac{1}{1+\big (\tau \big (\frac{1-(\beta _1)^q}{(\beta _1)^q}\big )^{\eta }\big )^{\frac{1}{\eta }}}} e^{i2\pi \root q \of {\frac{1}{1+\big (\tau \big (\frac{1-(\varpi _{\beta _{1}})^q}{(\varpi _{\beta _{1}})^q}\big )^{\eta }\big )^{\frac{1}{\eta }}}}} \end{array} \right) ,\\ \left( \begin{array}{c} \root q \of {1-\frac{1}{1+\big (\tau \big (\frac{(\gamma _1)^q}{1-(\gamma _1)^q}\big )^{\eta }\big )^{\frac{1}{\eta }}}} e^{i2\pi \root q \of {1-\frac{1}{1+\big (\tau \big (\frac{(\varpi _{\gamma _{1}})^q}{1-(\varpi _{\gamma _{1}})^q}\big )^{\eta }\big )^{\frac{1}{\eta }}}}} \end{array} \right) \end{array}\right) ; \tau \ge 0.$$

### Example 2

Consider two CTSFNs given by

$$\begin{aligned} \digamma _{1}=(0.8e^{i2\pi 0.81},0.5e^{i2\pi 0.52},0.9e^{i2\pi 0.93})\\ \digamma _{2}=(0.9e^{i2\pi 0.91},0.6e^{i2\pi 0.62},0.8e^{i2\pi 0.83}). \end{aligned}$$

Then for $\eta =1$ and $q=6$

$$\begin{aligned}&\digamma _1\oplus \digamma _2=\left( 0.918e^{i2\pi 0.926},0.477e^{i2\pi 0.496},0.773e^{i2\pi 0.808}\right) \\&\digamma _1\otimes \digamma _2=\left( 0.773e^{i2\pi 0.784},0.477e^{i2\pi 0.496},0.918e^{i2\pi 0.942}\right) \\&\tau \digamma =\left( 0.8e^{i2\pi 0.81},0.5e^{i2\pi 0.52},0.9e^{i2\pi 0.93}\right) , \tau =1\\&\digamma ^{\tau }=\left( 0.8e^{i2\pi 0.81},0.5e^{i2\pi 0.52},0.9e^{i2\pi 0.93}\right) . \end{aligned}$$

### Dombi weighted aggregation operators of CTSFNs

In this part, we introduce two operators called complex T-spherical Dombi fuzzy weighted arithmetic averaging (CTSDFWAA) operator and complex T-spherical Dombi fuzzy weighted geometric averaging (CTSDFWGA) operator. We also obtain some pivotal properties of the introduced operators.

### Complex T-spherical Dombi fuzzy weighted arithmetic averaging operator

#### Definition 13

Let ${\mathfrak {X}}$ be a universe and $\digamma _k=\Big (\alpha _ke^{i2\pi \varpi _{\alpha _k}},\beta _ke^{i2\pi \varpi _{\beta _k}},\gamma _ke^{i2\pi \varpi _{\gamma _k}}\Big )$ $(k=1,2,\ldots ,u)$ be a set of CTSFNs with weight vector ${\mathcal {W}}=({\mathcal {W}}_1,{\mathcal {W}}_2,\ldots ,{\mathcal {W}}_u)^T$, where ${\mathcal {W}}_k>0$, $\sum _{k=1}^{u}{\mathcal {W}}_k=1$. Then (CTSDFWAA) operator is defined by a mapping $\mathrm{CTSDFWAA}:\digamma ^u \rightarrow \digamma$, where

$$\begin{aligned} \mathrm{CTSDFWAA}(\digamma _1,\digamma _2,\ldots ,\digamma _u)=\bigoplus _{k=1}^u{\mathcal {W}}_k\digamma _k. \end{aligned}$$

#### Theorem 1

Let ${\mathfrak {X}}$ be a universe and $\digamma _k=\Big (\alpha _ke^{i2\pi \varpi _{\alpha _k}},\beta _ke^{i2\pi \varpi _{\beta _k}},\gamma _ke^{i2\pi \varpi _{\gamma _k}}\Big )$ $(k=1,2,\ldots ,u)$ be a set of CTSFNs of with weight vector ${\mathcal {W}}=({\mathcal {W}}_1,{\mathcal {W}}_2,\ldots ,{\mathcal {W}}_u)^T$, where $\mathcal {W_i}>0$ $(k=1,2,3,\ldots ,u)$ and $\sum _{k=1}^{u}{\mathcal {W}}_k=1$. Then, aggregated value of set using CTSDFWAA is a CTSFN defined as follows:

$$\begin{aligned} \mathrm{CTSDFWAA}(\digamma _1,\digamma _2,\ldots ,\digamma _u)=\bigoplus _{k=1}^u({\mathcal {W}}_k\digamma _k) \end{aligned}$$

$$\begin{aligned}&\qquad =\left( \begin{array}{c} \root q \of {1-\frac{1}{1+(\sum _{k=1}^{u}{\mathcal {W}}_k(\frac{\alpha _k^q}{1-\alpha _k^q})^{\eta })^{\frac{1}{\eta }}}} e^{i2\pi \root q \of {1-\frac{1}{1+\Big (\sum _{k=1}^{u}{\mathcal {W}}_k\big (\frac{(\varpi _{\alpha _k})^q}{1-(\varpi _{\alpha _k})^q}\big )^{\eta }\Big )^{\frac{1}{\eta }}}}},\\ \root q \of {\frac{1}{1+(\sum _{k=1}^{u}{\mathcal {W}}_k(\frac{1-\beta _k^q}{\beta _k^q})^{\eta })^{\frac{1}{\eta }}}} e^{i2\pi \root q \of {\frac{1}{1+\Big (\sum _{k=1}^{u}{\mathcal {W}}_k\big (\frac{1-(\varpi _{\beta _k})^q}{(\varpi _{\beta _k})^q}\big )^{\eta }\Big )^{\frac{1}{\eta }}}}}, \\ \root q \of {\frac{1}{1+(\sum _{k=1}^{u}{\mathcal {W}}_k(\frac{1-\gamma _k^q}{\gamma _k^q})^{\eta })^{\frac{1}{\eta }}}}e^{i2\pi \root q \of {\frac{1}{1+\Big (\sum _{k=1}^{u}{\mathcal {W}}_k\big (\frac{1-(\varpi _{\gamma _k})^q}{(\varpi _{\gamma _k})^q}\big )^{\eta }\Big )^{\frac{1}{\eta }}}}} \end{array}\right) . \end{aligned}$$

#### Proof

We can simply prove the theory using the mathematical induction method. Using Dombi operations of CTSFNs for $u = 2$, we have

$$\begin{aligned}&\mathrm{CTSDFWAA}(\digamma _1,\digamma _2)={\mathcal {W}}_{1}\digamma _1 \oplus {\mathcal {W}}_{2}\digamma _2\\&\quad =\left( \begin{array}{c} \root q \of {1-\frac{1}{1+({\mathcal {W}}_1(\frac{\alpha _1^q}{1-\alpha _1^q})^{\eta }+{\mathcal {W}}_2(\frac{\alpha _2^q}{1-\alpha _2^q})^{\eta })^{\frac{1}{\eta }}}} e^{i2\pi \root q \of {1-\frac{1}{1+\Big ({\mathcal {W}}_1\big (\frac{(\varpi _{\alpha _1})^q}{1-(\varpi _{\alpha _1})^q}\big )^{\eta }+ {\mathcal {W}}_2\big (\frac{(\varpi _{\alpha _2})^q}{1-(\varpi _{\alpha _2})^q}\big )^{\eta }\Big )^{\frac{1}{\eta }}}}},\\ \root q \of {\frac{1}{1+({\mathcal {W}}_1(\frac{\beta _1^q}{1-\beta _1^q})^{\eta }+{\mathcal {W}}_2(\frac{\beta _2^q}{1-\beta _2^q})^{\eta })^{\frac{1}{\eta }}}} e^{i2\pi \root q \of {\frac{1}{1+\Big ({\mathcal {W}}_1\big (\frac{(\varpi _{\beta _1})^q}{1-(\varpi _{\beta _1})^q}\big )^{\eta }+ {\mathcal {W}}_2\big (\frac{(\varpi _{\beta _2})^q}{1-(\varpi _{\beta _2})^q}\big )^{\eta }\Big )^{\frac{1}{\eta }}}}}, \\ \root q \of {\frac{1}{1+({\mathcal {W}}_1(\frac{\gamma _1^q}{1-\gamma _1^q})^{\eta }+{\mathcal {W}}_2(\frac{\gamma _2^q}{1-\gamma _2^q})^{\eta })^{\frac{1}{\eta }}}} e^{i2\pi \root q \of {\frac{1}{1+\Big ({\mathcal {W}}_1\big (\frac{(\varpi _{\gamma _1})^q}{1-(\varpi _{\gamma _1})^q}\big )^{\eta }+ {\mathcal {W}}_2\big (\frac{(\varpi _{\gamma _2})^q}{1-(\varpi _{\gamma _2})^q}\big )^{\eta }\Big )^{\frac{1}{\eta }}}}}, \end{array}\right) \\&\quad =\left( \begin{array}{c} \root q \of {1-\frac{1}{1+(\sum _{k=1}^{2}{\mathcal {W}}_k(\frac{\alpha _k^q}{1-\alpha _k^q})^{\eta })^{\frac{1}{\eta }}}} e^{i2\pi \root q \of {1-\frac{1}{1+\Big (\sum _{k=1}^{2}{\mathcal {W}}_k\big (\frac{(\varpi _{\alpha _k})^q}{1-(\varpi _{\alpha _k})^q}\big )^{\eta }\Big )^{\frac{1}{\eta }}}}},\\ \root q \of {\frac{1}{1+(\sum _{k=1}^{2}{\mathcal {W}}_k(\frac{1-\beta _k^q}{\beta _k^q})^{\eta })^{\frac{1}{\eta }}}} e^{i2\pi \root q \of {\frac{1}{1+\Big (\sum _{k=1}^{2}{\mathcal {W}}_k\big (\frac{1-(\varpi _{\beta _k})^q}{(\varpi _{\beta _k})^q})^{\eta }\big )^{\frac{1}{\eta }}}}}, \\ \root q \of {\frac{1}{1+(\sum _{k=1}^{2}{\mathcal {W}}_k(\frac{1-\gamma _k^q}{\gamma _k^q})^{\eta })^{\frac{1}{\eta }}}} e^{i2\pi \root q \of {\frac{1}{1+\Big (\sum _{k=1}^{2}{\mathcal {W}}_k\big (\frac{1-(\varpi _{\gamma _k})^q}{(\varpi _{\gamma _k})^q}\big )^{\eta }\Big )^{\frac{1}{\eta }}}}}. \end{array}\right) \end{aligned}$$

Assume that the equation holds, when $u = \sigma$, i.e.

$$\begin{aligned}&CTSDFWAA(\digamma _1,\digamma _2,\ldots ,\digamma _\sigma )={\mathcal {W}}_{1}\digamma _1 \oplus {\mathcal {W}}_{2}\digamma _2\oplus \ldots \oplus {\mathcal {W}}_{\sigma }\digamma _\sigma \\&=\left( \begin{array}{c} \root q \of {1-\frac{1}{1+(\sum _{k=1}^{\sigma }{\mathcal {W}}_k(\frac{\alpha _k^q}{1-\alpha _k^q})^{\eta })^{\frac{1}{\eta }}}} e^{i2\pi \root q \of {1-\frac{1}{1+\Big (\sum _{k=1}^{\sigma }{\mathcal {W}}_k\big (\frac{(\varpi _{\alpha _k})^q}{1-(\varpi _{\alpha _k})^q}\big )^{\eta }\Big )^{\frac{1}{\eta }}}}},\\ \root q \of {\frac{1}{1+(\sum _{k=1}^{\sigma }{\mathcal {W}}_k(\frac{1-\beta _k^q}{\beta _k^q})^{\eta })^{\frac{1}{\eta }}}} e^{i2\pi \root q \of {\frac{1}{1+\Big (\sum _{k=1}^{\sigma }{\mathcal {W}}_k\big (\frac{1-(\varpi _{\beta _k})^q}{(\varpi _{\beta _k})^q}\big )^{\eta }\Big )^{\frac{1}{\eta }}}}}, \\ \root q \of {\frac{1}{1+(\sum _{k=1}^{\sigma }{\mathcal {W}}_k(\frac{1-\gamma _k^q}{\gamma _k^q})^{\eta })^{\frac{1}{\eta }}}} e^{i2\pi \root q \of {\frac{1}{1+\Big (\sum _{k=1}^{\sigma }{\mathcal {W}}_k\big (\frac{1-(\varpi _{\gamma _k})^q}{(\varpi _{\gamma _k})^q}\big )^{\eta }\Big )^{\frac{1}{\eta }}}}} \end{array}\right) . \end{aligned}$$

If $u=\sigma +1$, then we have

$$\begin{aligned}&\mathrm{CTSDFWAA}(\digamma _1,\digamma _2,\ldots ,\digamma _\sigma )={\mathcal {W}}_{1}\digamma _1 \oplus {\mathcal {W}}_{2}\digamma _2\oplus \ldots \oplus {\mathcal {W}}_{\sigma }\digamma _\sigma \oplus {\mathcal {W}}_{\sigma +1}\digamma _{\sigma +1}\\&\quad =\left( \begin{array}{c} \root q \of {1-\frac{1}{1+(\sum _{k=1}^{\sigma }{\mathcal {W}}_k(\frac{\alpha _k^q}{1-\alpha _k^q})^{\eta })^{\frac{1}{\eta }}}} e^{i2\pi \root q \of {1-\frac{1}{1+\Big (\sum _{k=1}^{\sigma }{\mathcal {W}}_k\big (\frac{(\varpi _{\alpha _k})^q}{1-(\varpi _{\alpha _k})^q}\big )^{\eta }\Big )^{\frac{1}{\eta }}}}}, \\ \root q \of {\frac{1}{1+(\sum _{k=1}^{\sigma }{\mathcal {W}}_k(\frac{1-\beta _k^q}{\beta _k^q})^{\eta })^{\frac{1}{\eta }}}} e^{i2\pi \root q \of {\frac{1}{1+\Big (\sum _{k=1}^{\sigma }{\mathcal {W}}_k\big (\frac{1-(\varpi _{\beta _k})^q}{(\varpi _{\beta _k})^q})^{\eta }\big )^{\frac{1}{\eta }}}}}, \\ \root q \of {\frac{1}{1+(\sum _{k=1}^{\sigma }{\mathcal {W}}_k(\frac{1-\gamma _k^q}{\gamma _k^q})^{\eta })^{\frac{1}{\eta }}}} e^{i2\pi \root q \of {\frac{1}{1+\Big (\sum _{k=1}^{\sigma }{\mathcal {W}}_k\big (\frac{1-(\varpi _{\gamma _k})^q}{(\varpi _{\gamma _k})^q}\big )^{\eta }\Big )^{\frac{1}{\eta }}}}} \end{array}\right) \bigoplus (W_{\sigma +1}\digamma _{\sigma +1}) \\&\quad =\left( \begin{array}{c} \root q \of {1-\frac{1}{1+(\sum _{k=1}^{\sigma }{\mathcal {W}}_k(\frac{\alpha _k^q}{1-\alpha _k^q})^{\eta })^{\frac{1}{\eta }}}} e^{i2\pi \root q \of {1-\frac{1}{1+\Big (\sum _{k=1}^{\sigma }{\mathcal {W}}_k\big (\frac{(\varpi _{\alpha _k})^q}{1-(\varpi _{\alpha _k})^q}\big )^{\eta }\Big )^{\frac{1}{\eta }}}}}, \\ \root q \of {\frac{1}{1+(\sum _{k=1}^{\sigma }{\mathcal {W}}_k(\frac{1-\beta _k^q}{\beta _k^q})^{\eta })^{\frac{1}{\eta }}}} e^{i2\pi \root q \of {\frac{1}{1+\Big (\sum _{k=1}^{\sigma }{\mathcal {W}}_k\big (\frac{1-(\varpi _{\beta _k})^q}{(\varpi _{\beta _k})^q})^{\eta }\big )^{\frac{1}{\eta }}}}}, \\ \root q \of {\frac{1}{1+(\sum _{k=1}^{\sigma }{\mathcal {W}}_k(\frac{1-\gamma _k^q}{\gamma _k^q})^{\eta })^{\frac{1}{\eta }}}} e^{i2\pi \root q \of {\frac{1}{1+\Big (\sum _{k=1}^{\sigma }{\mathcal {W}}_k\big (\frac{1-(\varpi _{\gamma _k})^q}{(\varpi _{\gamma _k})^q}\big )^{\eta }\Big )^{\frac{1}{\eta }}}}} \end{array}\right) \bigoplus \\&\quad \times \left( \begin{array}{c} \root q \of {1-\frac{1}{1+({\mathcal {W}}_{\sigma +1}(\frac{\alpha _{\sigma +1}^q}{1-\alpha _{\sigma +1}^q})^{\eta })^{\frac{1}{\eta }}}} e^{i2\pi \root q \of {1-\frac{1}{1+\Big ({\mathcal {W}}_{\sigma +1}\big (\frac{(\varpi _{\alpha _{\sigma +1}})^q}{1-(\varpi _{\alpha _{\sigma +1}})^q}\big )^{\eta }\Big )^{\frac{1}{\eta }}}}},\\ \root q \of {\frac{1}{1+({\mathcal {W}}_{\sigma +1}(\frac{\beta _{\sigma +1}^q}{1-\beta _{\sigma +1}^q})^{\eta })^{\frac{1}{\eta }}}} e^{i2\pi \root q \of {\frac{1}{1+\Big ({\mathcal {W}}_{\sigma +1}\big (\frac{(\varpi _{\beta _{\sigma +1}})^q}{1-(\varpi _{\beta _{\sigma +1}})^q}\big )^{\eta }\Big )^{\frac{1}{\eta }}}}}, \\ \root q \of {\frac{1}{1+({\mathcal {W}}_{\sigma +1}(\frac{\gamma _{\sigma +1}^q}{1-\gamma _{\sigma +1}^q})^{\eta })^{\frac{1}{\eta }}}} e^{i2\pi \root q \of {\frac{1}{1+\Big ({\mathcal {W}}_{\sigma +1}\big (\frac{(\varpi _{\gamma _{\sigma +1}})^q}{1-(\varpi _{\gamma _{\sigma +1}})^q}\big )^{\eta }\Big )^{\frac{1}{\eta }}}}}, \end{array}\right) \\&\quad =\left( \begin{array}{c} \root q \of {1-\frac{1}{1+(\sum _{k=1}^{\sigma +1}{\mathcal {W}}_k(\frac{\alpha _k^q}{1-\alpha _k^q})^{\eta })^{\frac{1}{\eta }}}} e^{i2\pi \root q \of {1-\frac{1}{1+\Big (\sum _{k=1}^{\sigma +1}{\mathcal {W}}_k\big (\frac{(\varpi _{\alpha _k})^q}{1-(\varpi _{\alpha _k})^q}\big )^{\eta }\Big )^{\frac{1}{\eta }}}}},\\ \root q \of {\frac{1}{1+(\sum _{k=1}^{\sigma +1}{\mathcal {W}}_k(\frac{1-\beta _k^q}{\beta _k^q})^{\eta })^{\frac{1}{\eta }}}} e^{i2\pi \root q \of {\frac{1}{1+\Big (\sum _{k=1}^{\sigma +1}{\mathcal {W}}_k\big (\frac{1-(\varpi _{\beta _k})^q}{(\varpi _{\beta _k})^q})^{\eta }\big )^{\frac{1}{\eta }}}}}, \\ \root q \of {\frac{1}{1+(\sum _{k=1}^{\sigma +1}{\mathcal {W}}_k(\frac{1-\gamma _k^q}{\gamma _k^q})^{\eta })^{\frac{1}{\eta }}}} e^{i2\pi \root q \of {\frac{1}{1+\Big (\sum _{k=1}^{\sigma +1}{\mathcal {W}}_k\big (\frac{1-(\varpi _{\gamma _k})^q}{(\varpi _{\gamma _k})^q}\big )^{\eta }\Big )^{\frac{1}{\eta }}}}} \end{array}\right) . \end{aligned}$$

Hence, the theory 3.1 is true for $u=\sigma +1$. Then the equation is true for all $u \in \mathbb {N}.$ $\square$

#### Example 3

Consider three CTSFNs with weight vector $\mathbb {W}=(0.5,0.3,0.2)^{\tau }$ and operational parameter $\eta =1$ and $q=6$ given as follows:

$$\begin{aligned} \digamma _{1}=\left( 0.8e^{i2\pi 0.81},0.5e^{i2\pi 0.52},0.9e^{i2\pi 0.93}\right) ,\\ \digamma _{2}=\left( 0.9e^{i2\pi 0.91},0.6e^{i2\pi 0.62},0.8e^{i2\pi 0.83}\right) ,\\ \digamma _{3}=\left( 0.9e^{i2\pi 0.91},0.8e^{i2\pi 0.81},0.7e^{i2\pi 0.71}\right) . \end{aligned}$$

Using the CTSDFWAA operator, we can aggregate the three CTSFNs and find an aggregate value as shown below:

$$\begin{aligned} \mathrm{CTSDFWAA}\left( \digamma _{1},\digamma _{2},\digamma _{3})=\bigoplus _{k=1}^3({\mathcal {W}}_k\digamma _k \right) . \end{aligned}$$

$$\begin{aligned} =\left( \begin{array}{c} \root q \of {1-\frac{1}{1+(\sum _{k=1}^{3}{\mathcal {W}}_k(\frac{\alpha _k^q}{1-\alpha _k^q})^{\eta })^{\frac{1}{\eta }}}} e^{i2\pi \root q \of {1-\frac{1}{1+\Big (\sum _{k=1}^{u}{\mathcal {W}}_k\big (\frac{(\varpi _{\alpha _k})^q}{1-(\varpi _{\alpha _k})^q}\big )^{\eta }\Big )^{\frac{1}{\eta }}}}}, \\ \root q \of {\frac{1}{1+(\sum _{k=1}^{3}{\mathcal {W}}_k(\frac{1-\beta _k^q}{\beta _k^q})^{\eta })^{\frac{1}{\eta }}}} e^{i2\pi \root q \of {\frac{1}{1+\Big (\sum _{k=1}^{3}{\mathcal {W}}_k\big (\frac{1-(\varpi _{\beta _k})^q}{(\varpi _{\beta _k})^q}\big )^{\eta }\big )^{\frac{1}{\eta }}}}}, \\ \root q \of {\frac{1}{1+(\sum _{k=1}^{3}{\mathcal {W}}_k(\frac{1-\gamma _k^q}{\gamma _k^q})^{\eta })^{\frac{1}{\eta }}}} e^{i2\pi \root q \of {\frac{1}{1+\Big (\sum _{k=1}^{3}{\mathcal {W}}_k\big (\frac{1-(\varpi _{\gamma _k})^q}{(\varpi _{\gamma _k})^q}\big )^{\eta }\Big )^{\frac{1}{\eta }}}}} \end{array}\right) \end{aligned}$$

$$=(0.488e^{i2\pi 0.493},0.534e^{i2\pi 0.553},0.666e^{i2\pi 0.683}).$$

The values when $\eta \ne 1$ are shown in Table 1.

#### Theorem 2

(Idempotency) Let $\digamma _k=\Big (\alpha _ke^{i2\pi \varpi _{\alpha _k}},\beta _ke^{i2\pi \varpi _{\beta _k}},\gamma _ke^{i2\pi \varpi _{\gamma _k}}\Big )$ $(k=1,2,\ldots ,u)$ be a set of CTSFNs such that $\digamma _k=\digamma$. Then

$$\begin{aligned} \mathrm{CTSDFWAA}(\digamma _1,\digamma _2,\ldots ,\digamma _u)=\digamma . \end{aligned}$$

#### Proof

Assume that $\digamma _k = \digamma$ for all $k=1,2,\ldots ,u$. Using Eq. 3, we have

$$\begin{aligned} \mathrm{CTSDFWAA}(\digamma _1,\digamma _2,\ldots ,\digamma _u)=\bigoplus _{k=1}^u\left( {\mathcal {W}}_k\digamma _k\right) \end{aligned}$$

$$\begin{aligned} =\left( \begin{array}{c} \root q \of {1-\frac{1}{1+(\sum _{k=1}^{u}{\mathcal {W}}_k(\frac{\alpha _k^q}{1-\alpha _k^q})^{\eta })^{\frac{1}{\eta }}}} e^{i2\pi \root q \of {1-\frac{1}{1+\Big (\sum _{k=1}^{u}{\mathcal {W}}_k\big (\frac{(\varpi _{\alpha _k})^q}{1-(\varpi _{\alpha _k})^q}\big )^{\eta }\Big )^{\frac{1}{\eta }}}}},\\ \root q \of {\frac{1}{1+(\sum _{k=1}^{u}{\mathcal {W}}_k(\frac{1-\beta _k^q}{\beta _k^q})^{\eta })^{\frac{1}{\eta }}}} e^{i2\pi \root q \of {\frac{1}{1+\Big (\sum _{k=1}^{u}{\mathcal {W}}_k\big (\frac{1-(\varpi _{\beta _k})^q}{(\varpi _{\beta _k})^q})^{\eta }\big )^{\frac{1}{\eta }}}}}, \\ \root q \of {\frac{1}{1+(\sum _{k=1}^{u}{\mathcal {W}}_k(\frac{1-\gamma _k^q}{\gamma _k^q})^{\eta })^{\frac{1}{\eta }}}} e^{i2\pi \root q \of {\frac{1}{1+\Big (\sum _{k=1}^{u}{\mathcal {W}}_k\big (\frac{1-(\varpi _{\gamma _k})^q}{(\varpi _{\gamma _k})^q}\big )^{\eta }\Big )^{\frac{1}{\eta }}}}} \end{array}\right) \end{aligned}$$

$$\begin{aligned}&=\left( \begin{array}{c} \root q \of {1-\frac{1}{1+((\frac{\alpha _k^q}{1-\alpha _k^q})^{\eta })^{\frac{1}{\eta }}}} e^{i2\pi \root q \of {1-\frac{1}{1+\Big (\big (\frac{(\varpi _{\alpha _k})^q}{1-(\varpi _{\alpha _k})^q}\big )^{\eta }\Big )^{\frac{1}{\eta }}}}},\\ \root q \of {\frac{1}{1+((\frac{1-\beta _k^q}{\beta _k^q})^{\eta })^{\frac{1}{\eta }}}} e^{i2\pi \root q \of {\frac{1}{1+\Big (\big (\frac{1-(\varpi _{\beta _k})^q}{(\varpi _{\beta _k})^q})^{\eta }\big )^{\frac{1}{\eta }}}}}, \\ \root q \of {\frac{1}{1+((\frac{1-\gamma _k^q}{\gamma _k^q})^{\eta })^{\frac{1}{\eta }}}} e^{i2\pi \root q \of {\frac{1}{1+\Big (\big (\frac{1-(\varpi _{\gamma _k})^q}{(\varpi _{\gamma _k})^q}\big )^{\eta }\Big )^{\frac{1}{\eta }}}}} \end{array}\right) \\&=\left( \begin{array}{c} \root q \of {1-\frac{1}{1+(\frac{\alpha _k^q}{1-\alpha _k^q})}} e^{i2\pi \root q \of {1-\frac{1}{1+\big (\frac{(\varpi _{\alpha _k})^q}{1-(\varpi _{\alpha _k})^q}\big )}}},\\ \root q \of {\frac{1}{1+(\frac{1-\beta _k^q}{\beta _k^q})}} e^{i2\pi \root q \of {\frac{1}{1+\big (\frac{1-(\varpi _{\beta _k})^q}{(\varpi _{\beta _k})^q}}}}, \\ \root q \of {\frac{1}{1+(\frac{1-\gamma _k^q}{\gamma _k^q})}} e^{i2\pi \root q \of {\frac{1}{1+\big (\frac{1-(\varpi _{\gamma _k})^q}{(\varpi _{\gamma _k})^q}\big )}}} \end{array}\right) \\&=\Big (\alpha e^{i2\pi \varpi _{\alpha }},\beta e^{i2\pi \varpi _{\beta }},\gamma e^{i2\pi \varpi _{\gamma }}\Big )\\&=\digamma . \end{aligned}$$

$$\square $$

**Table 1:** When $$\eta \ne 1$$

$\eta$	CTSFNs
2	$(0.501e^{i2\pi 0.506},0.540e^{i2\pi 0.561},0.706e^{i2\pi 0.720})$
3	$(0.501e^{i2\pi 0.512},0.533e^{i2\pi 0.554},0.713e^{i2\pi 0.725})$
4	$(0.513e^{i2\pi 0.518},0.526e^{i2\pi 0.548},0.714e^{i2\pi 0.726})$
5	$(0.518e^{i2\pi 0.524},0.522e^{i2\pi 0.543},0.715e^{i2\pi 0.726})$

#### Theorem 3

(Monotonicity) Let $\digamma _k=\Big (\alpha _ke^{i2\pi \varpi _{\alpha _k}},\beta _ke^{i2\pi \varpi _{\beta _k}},\gamma _ke^{i2\pi \varpi _{\gamma _k}}\Big )$ and $\digamma _k'=\Big (\alpha _k'e^{i2\pi \varpi _{\alpha '_k}}, \beta _k'e^{i2\pi \varpi _{\beta '_k}},\gamma '_ke^{i2\pi \varpi _{\gamma '_k}}\Big )$ $(k=1,2,\ldots ,u)$ be two sets of CTSFNs. If $\alpha _k\le \alpha '_k, \beta _k\ge \beta '_k, \gamma _k\ge \gamma '_k$, $\varpi _{\alpha _k}\le \varpi _{\alpha '_k}, \varpi _{\beta _k}\ge \varpi _{\beta '_k},$ and $\varpi _{\gamma _k}\ge \varpi _{\gamma '_k}$ for all $k=1,2,\ldots ,u$. Then

$$\begin{aligned}&\mathrm{CTSDFWAA}(\digamma _1,\digamma _2,\ldots ,\digamma _u)\\&\quad \le \mathrm{CTSDFWAA}(\digamma '_1,\digamma '_2,\ldots ,\digamma '_u). \end{aligned}$$

#### Proof

Let CTSDFWAA$\digamma _k=\Big (\alpha _ke^{i2\pi \varpi _{\alpha _k}},\beta _ke^{i2\pi \varpi _{\beta _k}},\gamma _ke^{i2\pi \varpi _{\gamma _k}}\Big )$ and

CTSDFWAA $\digamma _k'=\Big (\alpha _k'e^{i2\pi \varpi _{\alpha '_k}},\beta _k'e^{i2\pi \varpi _{\beta '_k}},\gamma '_ke^{i2\pi \varpi _{\gamma '_k}}\Big ),k=\{1,2,3,\ldots ,u\}$ since $\alpha _{\digamma _{1}}\le \alpha _{\acute{\digamma _{1}}}$, $\frac{\alpha ^{q}_{\digamma _{1}}}{1-\alpha ^{q}_{\digamma _{1}}}\le \frac{\alpha ^{q}_{\acute{\digamma _{1}}}}{1-\alpha ^{q}_{\acute{\digamma _{1}}}}$. We show first that $\alpha \le \acute{\alpha }$. So, we have

$$\begin{aligned}&\left( \sum _{k=1}^{u}{\mathcal {W}}_k\left( \frac{\alpha ^{q}_{\digamma _{1}}}{1-\alpha ^{q}_{\digamma _{1}}}\right) ^{\eta }\right) ^{\frac{1}{\eta }}\\&\quad \le \left( \sum _{k=1}^{u}{\mathcal {W}}_k\left( \frac{\alpha ^{q}_{\acute{\digamma _{1}}}}{1-\alpha ^{q}_{\acute{\digamma _{1}}}}\right) ^{\eta }\right) ^{\frac{1}{\eta }}\\&\quad 1+ \left( \sum _{k=1}^{u}{\mathcal {W}}_k\left( \frac{\alpha ^{q}_{\digamma _{1}}}{1-\alpha ^{q}_{\digamma _{1}}}\right) ^{\eta }\right) ^{\frac{1}{\eta }}\\&\quad \le 1+\left( \sum _{k=1}^{u}{\mathcal {W}}_k\left( \frac{\alpha ^{q}_{\acute{\digamma _{1}}}}{1-\alpha ^{q}_{\acute{\digamma _{1}}}}\right) ^{\eta }\right) ^{\frac{1}{\eta }}\\&\quad \frac{1}{1+ \left( \sum _{k=1}^{u}{\mathcal {W}}_k\left( \frac{\alpha ^{q}_{\digamma _{1}}}{1-\alpha ^{q}_{\digamma _{1}}}\right) ^{\eta }\right) ^{\frac{1}{\eta }}}\\&\quad \ge \frac{1}{1+\left( \sum _{k=1}^{u}{\mathcal {W}}_k\left( \frac{\alpha ^{q}_{\acute{\digamma _{1}}}}{1-\alpha ^{q}_{\acute{\digamma _{1}}}}\right) ^{\eta }\right) ^{\frac{1}{\eta }}}\\&\quad 1-\frac{1}{1+ \left( \sum _{k=1}^{u}{\mathcal {W}}_k\left( \frac{\alpha ^{q}_{\digamma _{1}}}{1-\alpha ^{q}_{\digamma _{1}}}\right) ^{\eta }\right) ^{\frac{1}{\eta }}}\\&\quad \le 1-\frac{1}{1+\left( \sum _{k=1}^{u}{\mathcal {W}}_k\left( \frac{\alpha ^{q}_{\acute{\digamma _{1}}}}{1-\alpha ^{q}_{\acute{\digamma _{1}}}}\right) ^{\eta }\right) ^{\frac{1}{\eta }}}\\&\quad \root q \of {1-\frac{1}{1+ \left( \sum _{k=1}^{u}{\mathcal {W}}_k\left( \frac{\alpha ^{q}_{\digamma _{1}}}{1-\alpha ^{q}_{\digamma _{1}}}\right) ^{\eta }\right) ^{\frac{1}{\eta }}}}\\&\quad \le \root q \of {1-\frac{1}{1+\left( \sum _{k=1}^{u}{\mathcal {W}}_k \left( \frac{\alpha ^{q}_{\acute{\digamma _{1}}}}{1-\alpha ^{q}_{\acute{\digamma _{1}}}}\right) ^{\eta }\right) ^{\frac{1}{\eta }}}}. \end{aligned}$$

Hence, $\alpha \le \acute{\alpha }$ Similarly, it is easy to show that $\beta \le \acute{\beta }$ , $\gamma \le \acute{\gamma }$, $\varpi _{\alpha } \le \varpi _{\acute{\alpha }}$,$\varpi _{\beta } \le \varpi _{\acute{\beta }}$,$\varpi _{\gamma }\le \varpi _{\acute{\gamma }}$. Thus, the proof of the theorem is completed. $\square$

#### Theorem 4

(Boundedness) Let $\digamma _k=\Big (\alpha _ke^{i2\pi \varpi _{\alpha _k}},\beta _ke^{i2\pi \varpi _{\beta _k}}, \gamma _ke^{i2\pi \varpi _{\gamma _k}}\Big ),(k=1,2,3,\ldots ,u)$ a set of CTSFNs with $\digamma _{min}=min\left( \digamma _1,\digamma _2,\digamma _3,\ldots ,\digamma _u\right)$ and $\digamma _{max}=max\left( \digamma _1,\digamma _2,\digamma _3,\ldots ,\digamma _u\right)$. Then

$$\begin{aligned} \digamma _{min} \preceq CTSDFWAA\left( \digamma _1,\digamma _2,\digamma _3,\ldots ,\digamma _u\right) \preceq \digamma _{max}. \end{aligned}$$

#### Proof

Let

$$\begin{aligned} \digamma _{min}= & {} min\left( \digamma _1,\digamma _2,\digamma _3,\ldots ,\digamma _u\right) \\= & {} \Bigg (\alpha ^{-}_ke^{i2\pi \varpi ^{-}_{\alpha _k}},\beta ^{-}_ke^{i2\pi \varpi ^{-}_{\beta _k}},\gamma ^{+}_ke^{i2\pi \varpi ^{+}_{\gamma _k}}\Bigg ) \end{aligned}$$

and

$$\begin{aligned} \digamma _{max}= & {} max\left( \digamma _1,\digamma _2,\digamma _3,\ldots ,\digamma _u\right) \\= & {} \Bigg (\alpha ^{+}_ke^{i2\pi \varpi ^{+}_{\alpha _k}},\beta ^{-}_ke^{i2\pi \varpi ^{-}_{\beta _k}},\gamma ^{-}_ke^{i2\pi \varpi ^{-}_{\gamma _k}}\Bigg ).\end{aligned}$$

Therefore,

$$\begin{aligned} min(\alpha _{k})= & {} \alpha ^{-}_k,min(\beta _{k} )= \beta ^{-}_k,min(\gamma _{k} )= \gamma ^{-}_k,max(\alpha _{k} )\\= & {} \alpha ^{+}_k,max(\beta _{k})= \beta ^{+}_k,max(\gamma _{k} )= \gamma ^{+}_k \end{aligned}$$

$$\begin{aligned} min(\varpi _{\alpha _k} )= & {} \varpi ^{-}_{\alpha _k},min(\varpi _{\beta _k})= \varpi ^{-}_{\beta _k},min(\varpi _{\gamma _k} )\\= & {} \varpi ^{-}_{\gamma _k},max(\varpi _{\alpha _k} ) = \varpi ^{+}_{\alpha _k},max(\varpi _{\beta _k} )\\= & {} \varpi ^{+}_{\beta _k},max(\varpi _{\gamma _k} )= \varpi ^{+}_{\gamma _k}. \end{aligned}$$

The inequality for amplitude term of membership grade is given as follows:

$$\begin{aligned}&\root q \of {1-\frac{1}{1+(\sum _{k=1}^{3}{\mathcal {W}}_k(\frac{\alpha _k^{-q}}{1-\alpha _k^{-q}})^{\eta })^{\frac{1}{\eta }}}}\\&\quad \le \root q \of {1-\frac{1}{1+(\sum _{k=1}^{3}{\mathcal {W}}_k(\frac{\alpha _k^q}{1-\alpha _k^q})^{\eta })^{\frac{1}{\eta }}}}\\&\quad \le \root q \of {1-\frac{1}{1+(\sum _{k=1}^{3}{\mathcal {W}}_k(\frac{\alpha _k^{+q}}{1-\alpha _k^{+q}})^{\eta })^{\frac{1}{\eta }}}}. \end{aligned}$$

Similarly, the inequality for phase term of membership grade is given as follows:

$$\begin{aligned}&\root q \of {1-\frac{1}{1+\Big (\sum _{k=1}^{u}{\mathcal {W}}_k\big (\frac{(\varpi _{\alpha _k})^{-q}}{1-(\varpi _{\alpha _k})^{-q}}\big )^{\eta }\Big )^{\frac{1}{\eta }}}}\\&\quad \le \root q \of {1-\frac{1}{1+\Big (\sum _{k=1}^{u}{\mathcal {W}}_k\big (\frac{(\varpi _{\alpha _k})^q}{1-(\varpi _{\alpha _k})^q}\big )^{\eta }\Big )^{\frac{1}{\eta }}}}\\&\quad \le \root q \of {1-\frac{1}{1+\Big (\sum _{k=1}^{u}{\mathcal {W}}_k\big (\frac{(\varpi _{\alpha _k})^{+q}}{1-(\varpi _{\alpha _k})^{+q}}\big )^{\eta }\Big )^{\frac{1}{\eta }}}}. \end{aligned}$$

In a similar manner, we can get the results for the amplitude and phase terms of abstinence and non-membership grades. Thus,

$$\begin{aligned} \digamma _\mathrm{min} \preceq \mathrm{CTSDFWAA}\left( \digamma _1,\digamma _2,\digamma _3,\ldots ,\digamma _u\right) \preceq \digamma _\mathrm{max}. \end{aligned}$$

$$\square $$

### Complex T-spherical Dombi fuzzy weighted geometric averaging operator

#### Definition 14

Let $\digamma _k=\Big (\alpha _ke^{i2\pi \varpi _{\alpha _k}},\beta _ke^{i2\pi \varpi _{\beta _k}},\gamma _ke^{i2\pi \varpi _{\gamma _k}}\Big )$ $(k=1,2,\ldots ,u)$ be a set of CTSFNs of with weight vector ${\mathcal {W}}=({\mathcal {W}}_1,{\mathcal {W}}_2,\ldots ,{\mathcal {W}}_u)^T$, where ${\mathcal {W}}_k>0$, $\sum _{k=1}^{u}{\mathcal {W}}_k=1$. Then (CTSDFWGA) operator is defined by a mapping CTSDFWGA$:\digamma ^u \rightarrow \digamma$, where

$$\begin{aligned} \mathrm{CTSDFWGA}\left( \digamma _1,\digamma _2,\ldots ,\digamma _u\right) =\bigotimes _{k=1}^u\digamma _k^{{\mathcal {W}}_k}. \end{aligned}$$

#### Theorem 5

If $\digamma _k=\Big (\alpha _ke^{i2\pi \varpi _{\alpha _k}},\beta _ke^{i2\pi \varpi _{\beta _k}},\gamma _ke^{i2\pi \varpi _{\gamma _k}}\Big )$ $(k=1,2,\ldots ,u)$ be a set of CTSFNs of with weight vector ${\mathcal {W}}=({\mathcal {W}}_1,{\mathcal {W}}_2,\ldots ,{\mathcal {W}}_u)^T$, where ${\mathcal {W}}_k>0$, $\sum _{k=1}^{u}{\mathcal {W}}_k=1$. Then (CTSDFWGA) operator the clumped value of these *CTSFNs* is again a *CTSFN*. This clumped value can be obtained by the following formula:

$$\begin{aligned} \mathrm{CTSDFWGA}(\digamma _1,\digamma _2,\ldots ,\digamma _u)=\bigotimes _{k=1}^u\digamma _k^{{\mathcal {W}}_k} \end{aligned}$$

$$\begin{aligned} =\left( \begin{array}{c} \root q \of {\frac{1}{1+(\sum _{k=1}^{u}{\mathcal {W}}_k(\frac{1-\alpha _k^q}{\alpha _k^q})^{\eta })^{\frac{1}{\eta }}}}e^{i2\pi \root q \of {\frac{1}{1+\Big (\sum _{k=1}^{u}{\mathcal {W}}_k\big (\frac{1-(\varpi _{\alpha _k})^q}{(\varpi _{\alpha _k})^q}\big )^{\eta }\Big )^{\frac{1}{\eta }}}}} , \\ \root q \of {\frac{1}{1+(\sum _{k=1}^{u}{\mathcal {W}}_k(\frac{1-\beta _k^q}{\beta _k^q})^{\eta })^{\frac{1}{\eta }}}} e^{i2\pi \root q \of {\frac{1}{1+\Big (\sum _{k=1}^{u}{\mathcal {W}}_k\big (\frac{1-(\varpi _{\beta _k})^q}{(\varpi _{\beta _k})^q}\big )^{\eta }\Big )^{\frac{1}{\eta }}}}}, \\ \root q \of {1-\frac{1}{1+(\sum _{k=1}^{u}{\mathcal {W}}_k(\frac{\gamma _k^q}{1-\gamma _k^q})^{\eta })^{\frac{1}{\eta }}}} e^{i2\pi \root q \of {1-\frac{1}{1+\Big (\sum _{k=1}^{u}{\mathcal {W}}_k\big (\frac{(\varpi _{\gamma _k})^q}{1-(\varpi _{\gamma _k})^q}\big )^{\eta }\Big )^{\frac{1}{\eta }}}}}. \end{array}\right) \end{aligned}$$

#### Proof

The proof can be made by similar way to the proof of Theorem 9. $\square$

#### Example 4

Let us consider three CTSFNs given as follows:

$$\begin{aligned} \digamma _{1}=\left( 0.8e^{i2\pi 0.81},0.5e^{i2\pi 0.52},0.9e^{i2\pi 0.93}\right) ,\\ \digamma _{2}=\left( 0.9e^{i2\pi 0.91},0.6e^{i2\pi 0.62},0.8e^{i2\pi 0.83}\right) ,\\ \digamma _{3}=\left( 0.9e^{i2\pi 0.91},0.8e^{i2\pi 0.81},0.7e^{i2\pi 0.71}\right) \end{aligned}$$

with the weight vector $\mathbb {W}=(0.5,0.3,0.2)^{\tau }$ and operational parameter $\eta =1$ and $q=6$. Using the CTSDFWGA operator, we can aggregate the three CTSFNs and find a clumped value as given below:

$$\begin{aligned}\mathrm{CTSDFWGA}(\digamma _1,\digamma _2,\ldots ,\digamma _u)=\bigotimes _{k=1}^u\digamma _k^{{\mathcal {W}}_k} \end{aligned}$$

$$\begin{aligned} \quad =\left( \begin{array}{c} \root q \of {\frac{1}{1+\left( \sum _{k=1}^{u}{\mathcal {W}}_k\left( \frac{1-\alpha _k^q}{\alpha _k^q}\right) ^{\eta }\right) ^{\frac{1}{\eta }}}}e^{i2\pi \root q \of {\frac{1}{1+\Bigg (\sum _{k=1}^{u}{\mathcal {W}}_k\bigg (\frac{1-(\varpi _{\alpha _k})^q}{(\varpi _{\alpha _k})^q}\bigg )^{\eta }\Bigg )^{\frac{1}{\eta }}}}} , \\ \root q \of {\frac{1}{1+\left( \sum _{k=1}^{u}{\mathcal {W}}_k\left( \frac{1-\beta _k^q}{\beta _k^q}\right) ^{\eta }\right) ^{\frac{1}{\eta }}}} e^{i2\pi \root q \of {\frac{1}{1+\Bigg (\sum _{k=1}^{u}{\mathcal {W}}_k\bigg (\frac{1-(\varpi _{\beta _k})^q}{(\varpi _{\beta _k})^q}\bigg )^{\eta }\Bigg )^{\frac{1}{\eta }}}}}, \\ \root q \of {1-\frac{1}{1+\left( \sum _{k=1}^{u}{\mathcal {W}}_k\left( \frac{\gamma _k^q}{1-\gamma _k^q}\right) ^{\eta }\right) ^{\frac{1}{\eta }}}} e^{i2\pi \root q \of {1-\frac{1}{1+\Bigg (\sum _{k=1}^{u}{\mathcal {W}}_k\bigg (\frac{(\varpi _{\gamma _k})^q}{1-(\varpi _{\gamma _k})^q}\bigg )^{\eta }\Bigg )^{\frac{1}{\eta }}}}} \end{array}\right) \end{aligned}$$

$$=\left( 0.788e^{i2\pi 0.801},0.534e^{i2\pi 0.553},0.443e^{i2\pi 0.461}\right) $$.

The values when $\eta \ne 1$ are shown in Table 2.

#### Theorem 6

(Idempotency) Let $\digamma _k=\Bigg (\alpha _ke^{i2\pi \varpi _{\alpha _k}},\beta _ke^{i2\pi \varpi _{\beta _k}},\gamma _ke^{i2\pi \varpi _{\gamma _k}}\Bigg )$ $(k=1,2,\ldots ,u)$ be a set of CTSFNs such that $\digamma _k=\digamma$. Then

$$\begin{aligned} \mathrm{CTSDFWGA}\left( \digamma _1,\digamma _2,\ldots ,\digamma _u\right) =\digamma . \end{aligned}$$

#### Proof

The proof is similar to the proof of Theorem 2. $\square$

#### Theorem 7

(Monotonicity) Let $\digamma _k=\Big (\alpha _ke^{i2\pi \varpi _{\alpha _k}},\beta _ke^{i2\pi \varpi _{\beta _k}},\gamma _ke^{i2\pi \varpi _{\gamma _k}}\Big )$ and $\digamma _k'=\Big (\alpha _k'e^{i2\pi \varpi _{\alpha '_k}}, \beta _k'.e^{i2\pi \varpi _{\beta '_k}},\gamma '_ke^{i2\pi \varpi _{\gamma '_k}}\Big )$ $(k=1,2,\ldots ,u)$ be two sets of CTSFNs. If $\alpha _k\le \alpha '_k, \beta _k\ge \beta '_k, \gamma _k\ge \gamma '_k$, $\varpi _{\alpha _k}\le \varpi _{\alpha '_k}, \varpi _{\beta _k}\ge \varpi _{\beta '_k},$ and $\varpi _{\gamma _k}\ge \varpi _{\gamma '_k}$ for all *k*, then

$$\begin{aligned}&\mathrm{CTSDFWGA}\left( \digamma _1,\digamma _2,\ldots ,\digamma _u\right) \\&\quad \le \mathrm{CTSDFWGA}\left( \digamma '_1,\digamma '_2,\ldots ,\digamma '_u\right) . \end{aligned}$$

#### Proof

The proof can be made by the similar way to Theorem 3. $\square$

#### Theorem 8

(Boundedness) Let $\digamma _k=\Big (\alpha _ke^{i2\pi \varpi _{\alpha _k}},\beta _ke^{i2\pi \varpi _{\beta _k}}, \gamma _ke^{i2\pi \varpi _{\gamma _k}}\Big ), (k=1,2,3,\ldots ,u)$ be a set of CTSFNs with $\digamma _{min}=min(\digamma _1,\digamma _2,\digamma _3,\ldots ,\digamma _u)$ and $\digamma _{max}=max(\digamma _1,\digamma _2,\digamma _3,\ldots ,\digamma _u)$. Then

$$\begin{aligned} \digamma _\mathrm{min} \preceq \mathrm{CTSDFWGA}\left( \digamma _1,\digamma _2,\digamma _3,\ldots ,\digamma _u\right) \preceq \digamma _\mathrm{max}. \end{aligned}$$

#### Proof

The proof can be made by the similar way to proof of Theorem 4. $\square$

**Table 2:** When $$\eta \ne 1$$

$\eta$	CTSFNs
2	$\left( 0.855e^{i2\pi 0.869},0.540e^{i2\pi 0.561},0.476e^{i2\pi 0.505}\right)$
3	$\left( 0.865e^{i2\pi 0.880},0.533e^{i2\pi 0.554},0.506e^{i2\pi 0.546}\right)$
4	$\left( 0.864e^{i2\pi 0.878},0.526e^{i2\pi 0.548},0.535e^{i2\pi 0.588}\right)$
5	$\left( 0.861e^{i2\pi 0.875},0.522e^{i2\pi 0.543},0.875e^{i2\pi 0.629}\right)$

### Dombi ordered weighted aggregation operators of CTSFNs

In this part, we propose two operators, namely, complex T-spherical Dombi fuzzy ordered weighted arithmetic averaging (CTSDFOWAA) operator and complex T-spherical Dombi fuzzy weighted ordered geometric averaging (CTSDFOWGA) operator. Moreover, we also discuss some pivotal properties of these operators.

### Complex T-spherical Dombi fuzzy ordered weighted arithmetic averaging operator

#### Definition 15

Let $\digamma _k=\Big (\alpha _ke^{i2\pi \varpi _{\alpha _k}},\beta _ke^{i2\pi \varpi _{\beta _k}},\gamma _ke^{i2\pi \varpi _{\gamma _k}}\Big )$ $(k=1,2,\ldots ,u)$ be a set of CTSFNs of with weight vector ${\mathcal {W}}=({\mathcal {W}}_1,{\mathcal {W}}_2,\ldots ,{\mathcal {W}}_u)^T$, where ${\mathcal {W}}_k>0$, $\sum _{k=1}^{u}{\mathcal {W}}_k=1$. Then (CTSDFOWAA) operator is defined by a mapping

$$\mathrm{CTSDFOWAA}:\digamma ^u \rightarrow \digamma $$, where

$$\begin{aligned} \mathrm{CTSDFOWAA}\left( \digamma _1,\digamma _2,\ldots ,\digamma _u\right) =\bigoplus _{k=1}^u{\mathcal {W}}_k\digamma _{\sigma (k)} \end{aligned}$$

and $\left( \sigma _{1},\sigma _{2},\ldots ,\sigma _{u}\right)$ are the permutations of $\sigma (k)$ having the condition $|\digamma _{\sigma (k-1)}| \ge |\digamma _{\sigma (k)}|$ for all $(k=1,2,\ldots ,u).$

#### Theorem 9

If $\digamma _k=\Big (\alpha _ke^{i2\pi \varpi _{\alpha _k}},\beta _ke^{i2\pi \varpi _{\beta _k}},\gamma _ke^{i2\pi \varpi _{\gamma _k}}\Big )$ $(k=1,2,\ldots ,u)$ be a set of CTSFNs of with weight vector ${\mathcal {W}}=({\mathcal {W}}_1,{\mathcal {W}}_2,\ldots ,{\mathcal {W}}_u)^T$, where ${\mathcal {W}}>0$ and $\sum _{k=1}^{u}{\mathcal {W}}_k=1$.

Then aggregated value of set using CTSDFOWAA is a CTSFN defined as follows:

$$\begin{aligned}&\mathrm{CTSDFOWAA}\left( \digamma _1,\digamma _2,\ldots ,\digamma _u\right) =\bigoplus _{k=1}^u\left( {\mathcal {W}}_k\digamma _{\sigma (k)}\right) \\&\quad =\left( \begin{array}{c} \root q \of {1-\frac{1}{1+\left( \sum _{k=1}^{u}{\mathcal {W}}_k\left( \frac{\alpha _{\sigma (k)}^q}{1-\alpha _{\sigma (k)}^q}\right) ^{\eta }\right) ^{\frac{1}{\eta }}}} e^{i2\pi \root q \of {1-\frac{1}{1+\Bigg (\sum _{k=1}^{u}{\mathcal {W}}_k\bigg (\frac{(\varpi _{\alpha _{\sigma (k)}})^q}{1-(\varpi _{\alpha _{\sigma (k)}})^q}\bigg )^{\eta }\Bigg )^{\frac{1}{\eta }}}}},\\ \root q \of {\frac{1}{1+\left( \sum _{k=1}^{u}{\mathcal {W}}_k\left( \frac{1-\beta _{\sigma (k)}^q}{\beta _{\sigma (k)}^q}\right) ^{\eta }\right) ^{\frac{1}{\eta }}}} e^{i2\pi \root q \of {\frac{1}{1+\Bigg (\sum _{k=1}^{u}{\mathcal {W}}_k\bigg (\frac{1-(\varpi _{\beta _{\sigma (k)}})^q}{(\varpi _{\beta _{\sigma (k)}})^q}\bigg )^{\eta }\Bigg )^{\frac{1}{\eta }}}}}, \\ \root q \of {\frac{1}{1+\left( \sum _{k=1}^{u}{\mathcal {W}}_k\left( \frac{1-\gamma _{\sigma (k)}^q}{\gamma _{\sigma (k)}^q}\right) ^{\eta }\right) ^{\frac{1}{\eta }}}}e^{i2\pi \root q \of {\frac{1}{1+\Bigg (\sum _{k=1}^{u}{\mathcal {W}}_k\bigg (\frac{1-(\varpi _{\gamma _{\sigma (k)}})^q}{\left( \varpi _{\gamma _{\sigma (k)}}\right) ^q}\bigg )^{\eta }\Bigg )^{\frac{1}{\eta }}}}} \end{array}\right) . \end{aligned}$$

#### Example 5

Let us consider three CTSFNs given as follows:

$$\begin{aligned} \digamma _{1}=\left( 0.9e^{i2\pi 0.91},0.7e^{i2\pi 0.62},0.8e^{i2\pi 0.83}\right) ,\\ \digamma _{2}=\left( 0.9e^{i2\pi 0.91},0.8e^{i2\pi 0.81},0.3e^{i2\pi 0.50}\right) ,\\ \digamma _{3}=\left( 0.8e^{i2\pi 0.81},0.5e^{i2\pi 0.52},0.4e^{i2\pi 0.67}\right) \end{aligned}$$

having the weight vector ${\mathcal {W}}=(0.5,0.3,0.2)^{\tau }$ with operational parameter $\eta =1$ and $q=6$. Using the CTSDFOWAA operator, we can aggregate the three CTSFNs. We can find the values of the score function of this CTSFNs

$$\begin{aligned} \Omega (\digamma _{1})=0.584 , \Omega (\digamma _{2})=0.635 , \Omega (\digamma _{3})=0.604. \end{aligned}$$

Hence, $\Omega (\digamma _{2}) \ge \Omega (\digamma _{3}) \ge \Omega (\digamma _{1})$, we will also find that

$$\begin{aligned}&\digamma _{\sigma 1}=\digamma _{2}=\left( 0.9e^{i2\pi 0.91},0.8e^{i2\pi 0.81},0.3e^{i2\pi 0.50}\right) ,\\&\quad \digamma _{\sigma 2}=\digamma _{3}=\left( 0.8e^{i2\pi 0.81},0.5e^{i2\pi 0.52},0.4e^{i2\pi 0.67}\right) ,\\&\quad \digamma _{\sigma 3}=\digamma _{1}=\left( 0.9e^{i2\pi 0.91},0.7e^{i2\pi 0.62},0.8e^{i2\pi 0.83}\right) .\\&\quad \mathrm{CTSDFOWAA}\left( \digamma _1,\digamma _2,\digamma _3\right) =\bigoplus _{k=1}^3\left( {\mathcal {W}}_k\digamma _{\sigma (k)}\right) \end{aligned}$$

$$\begin{aligned}&\quad =\left( \begin{array}{c} \root q \of {1-\frac{1}{1+\left( \sum _{k=1}^{3}{\mathcal {W}}_k\left( \frac{\alpha _{\sigma (k)}^q}{1-\alpha _{\sigma (k)}^q}\right) ^{\eta }\right) ^{\frac{1}{\eta }}}} e^{i2\pi \root q \of {1-\frac{1}{1+\Bigg (\sum _{k=1}^{u}{\mathcal {W}}_k\bigg (\frac{(\varpi _{\alpha _{\sigma (k)}})^q}{1-(\varpi _{\alpha _{\sigma (k)}})^q}\bigg )^{\eta }\Bigg )^{\frac{1}{\eta }}}}},\\ \root q \of {\frac{1}{1+\left( \sum _{k=1}^{3}{\mathcal {W}}_k\left( \frac{1-\beta _{\sigma (k)}^q}{\beta _{\sigma (k)}^q}\right) ^{\eta }\right) ^{\frac{1}{\eta }}}} e^{i2\pi \root q \of {\frac{1}{1+\Bigg (\sum _{k=1}^{3}{\mathcal {W}}_k\bigg (\frac{1-\left( \varpi _{\beta _{\sigma (k)}}\right) ^q}{(\varpi _{\beta _{\sigma (k)}})^q}\bigg )^{\eta }\Bigg )^{\frac{1}{\eta }}}}}, \\ \root q \of {\frac{1}{1+\left( \sum _{k=1}^{3}{\mathcal {W}}_k\left( \frac{1-\gamma _{\sigma (k)}^q}{\gamma _{\sigma (k)}^q}\right) ^{\eta }\right) ^{\frac{1}{\eta }}}}e^{i2\pi \root q \of {\frac{1}{1+\Bigg (\sum _{k=1}^{u}{\mathcal {W}}_k\bigg (\frac{1-(\varpi _{\gamma _{\sigma (k)}})^q}{\left( \varpi _{\gamma _{\sigma (k)}}\right) ^q}\bigg )^{\eta }\Bigg )^{\frac{1}{\eta }}}}} \end{array}\right) \end{aligned}$$

$$=\left( 0.488e^{i2\pi 0.493},0.489e^{i2\pi 0.508},0.682e^{i2\pi 0.696}\right) .$$

#### Theorem 10

(Idempotency) Let $\digamma _k=\Bigg (\alpha _ke^{i2\pi \varpi _{\alpha _k}},\beta _ke^{i2\pi \varpi _{\beta _k}},\gamma _ke^{i2\pi \varpi _{\gamma _k}}\Bigg )$ $(k=1,2,\ldots ,u)$ be a set of CTSFNs such that $\digamma _k=\digamma$. Then

$$\begin{aligned} \mathrm{CTSDFOWAA}\left( \digamma _1,\digamma _2,\ldots ,\digamma _u \right) =\digamma . \end{aligned}$$

#### Theorem 11

(Monotonicity) Let $\digamma _k=\Big (\alpha _ke^{i2\pi \varpi _{\alpha _k}},\beta _ke^{i2\pi \varpi _{\beta _k}},\gamma _ke^{i2\pi \varpi _{\gamma _k}}\Big )$ and $\digamma _k'=\Big (\alpha _k'e^{i2\pi \varpi _{\alpha '_k}}, \beta _k'.e^{i2\pi \varpi _{\beta '_k}},\gamma '_ke^{i2\pi \varpi _{\gamma '_k}}\Big )$ $(k=1,2,\ldots ,u)$ be two sets of CTSFNs. If $\alpha _k\le \alpha '_k, \beta _k\ge \beta '_k, \gamma _k\ge \gamma '_k$, $\varpi _{\alpha _k}\le \varpi _{\alpha '_k}, \varpi _{\beta _k}\ge \varpi _{\beta '_k},$ and $\varpi _{\gamma _k}\ge \varpi _{\gamma '_k}$ for all *k*. Then

$$\begin{aligned}&CTSDFOWAA(\digamma _1,\digamma _2,\ldots ,\digamma _u)\\&\quad \preceq CTSDFOWAA(\digamma '_1,\digamma '_2,\ldots ,\digamma '_u). \end{aligned}$$

#### Theorem 12

(Boundedness) Let $\digamma _k=\Big (\alpha _ke^{i2\pi \varpi _{\alpha _k}},\beta _ke^{i2\pi \varpi _{\beta _k}},\gamma _ke^{i2\pi \varpi _{\gamma _k}}\Big ),k=\{1,2,3,\ldots ,u\}$ a set of CTSFNs with $\digamma _{min}=min(\digamma _1,\digamma _2,\digamma _3,\ldots ,\digamma _u)$ and $\digamma _{max}=max(\digamma _1,\digamma _2,\digamma _3,\ldots ,\digamma _u)$. Then

$$\begin{aligned} \digamma _{min} \preceq CTSDFOWAA(\digamma _1,\digamma _2,\digamma _3,\ldots ,\digamma _u) \preceq \digamma _{max}. \end{aligned}$$

### Complex T-spherical Dombi fuzzy ordered weighted geometric averaging operator

#### Definition 16

Let $\digamma _k=\Big (\alpha _ke^{i2\pi \varpi _{\alpha _k}},\beta _ke^{i2\pi \varpi _{\beta _k}},\gamma _ke^{i2\pi \varpi _{\gamma _k}}\Big )$ $(k=1,2,\ldots ,u)$ be a set of CTSFNs of with weight vector ${\mathcal {W}}=({\mathcal {W}}_1,{\mathcal {W}}_2,\ldots ,{\mathcal {W}}_u)^T$, where ${\mathcal {W}}_k>0$, $\sum _{k=1}^{u}{\mathcal {W}}_k=1$. Then (CTSDFOWGA) operator is defined by a mapping $CTSDFOWGA:\digamma _u \rightarrow \digamma$, where

$$\begin{aligned} \mathrm{CTSDFOWGA}\left( \digamma _1,\digamma _2,\ldots ,\digamma _u\right) =\bigotimes _{k=1}^u\digamma _{\sigma (k)}^{{\mathcal {W}}_k} \end{aligned}$$

and $\left( \sigma _{1},\sigma _{2},\ldots ,\sigma _{u}\right)$ are the permutations of $\sigma (k)$ having the condition $|\digamma _{\sigma (k-1)}| \ge |\digamma _{\sigma (k)}|$ for all $(k=1,2,\ldots ,u)$

#### Theorem 13

If $\digamma _k=\Bigg (\alpha _ke^{i2\pi \varpi _{\alpha _k}},\beta _ke^{i2\pi \varpi _{\beta _k}},\gamma _ke^{i2\pi \varpi _{\gamma _k}}\Bigg )$ $(k=1,2,\ldots ,u)$ be a set of CTSFNs of with weight vector ${\mathcal {W}}=({\mathcal {W}}_1,{\mathcal {W}}_2,\ldots ,{\mathcal {W}}_u)^T$, where ${\mathcal {W}}_k>0$, $\sum _{k=1}^{u}{\mathcal {W}}_k=1$. Then (CTSDFWGA) operator the clumped value of these *CTSFNs* is again a *CTSFN*. This clumped value can be obtained by the following formula:

$$\begin{aligned}\mathrm{CTSDFOWGA}\left( \digamma _1,\digamma _2,\ldots ,\digamma _u\right) =\bigotimes _{k=1}^u\digamma _{\sigma (k)}^{{\mathcal {W}}_k} \end{aligned}$$

$$\begin{aligned} =\left( \begin{array}{c} \root q \of {\frac{1}{1+\left( \sum _{k=1}^{u}{\mathcal {W}}_k\left( \frac{1-\alpha _{\sigma (k)}^q}{\alpha _{\sigma (k)}^q}\right) ^{\eta }\right) ^{\frac{1}{\eta }}}}e^{i2\pi \root q \of {\frac{1}{1+\Bigg (\sum _{k=1}^{u}{\mathcal {W}}_k\bigg (\frac{1-\left( \varpi _{\alpha _{\sigma (k)}}\right) ^q}{\left( \varpi _{\alpha _{\sigma (k)}}\right) ^q}\bigg )^{\eta }\Bigg )^{\frac{1}{\eta }}}}} , \\ \root q \of {\frac{1}{1+\left( \sum _{k=1}^{u}{\mathcal {W}}_k\left( \frac{1-\beta _{\sigma (k)}^q}{\beta _{\sigma (k)}^q}\right) ^{\eta }\right) ^{\frac{1}{\eta }}}} e^{i2\pi \root q \of {\frac{1}{1+\Bigg (\sum _{k=1}^{u}{\mathcal {W}}_k\bigg (\frac{1-(\varpi _{\beta _{\sigma (k)}})^q}{(\varpi _{\beta _{\sigma (k)}})^q}\bigg )^{\eta }\Bigg )^{\frac{1}{\eta }}}}}, \\ \root q \of {1-\frac{1}{1+\left( \sum _{k=1}^{u}{\mathcal {W}}_k\left( \frac{\gamma _{\sigma (k)}^q}{1-\gamma _{\sigma (k)}^q}\right) ^{\eta }\right) ^{\frac{1}{\eta }}}} e^{i2\pi \root q \of {1-\frac{1}{1+\Bigg (\sum _{k=1}^{u}{\mathcal {W}}_k\bigg (\frac{(\varpi _{\gamma _{\sigma (k)}})^q}{1-(\varpi _{\gamma _{\sigma (k)}})^q}\bigg )^{\eta }\Bigg )^{\frac{1}{\eta }}}}}. \end{array}\right) \end{aligned}$$

#### Example 6

Consider CTSFNs given in Example 3.4. Then

$$\begin{aligned}\mathrm{CTSDFOWGA}\left( \digamma _1,\digamma _2,\digamma _3\right) =\bigotimes _{k=1}^3\digamma _{\sigma (k)}^{{\mathcal {W}}_k} \end{aligned}$$

$$\begin{aligned} =\left( \begin{array}{c} \root q \of {\frac{1}{1+\left( \sum _{k=1}^{u}{\mathcal {W}}_k\left( \frac{1-\alpha _{\sigma (k)}^q}{\alpha _{\sigma (k)}^q}\right) ^{\eta }\right) ^{\frac{1}{\eta }}}}e^{i2\pi \root q \of {\frac{1}{1+\Bigg (\sum _{k=1}^{u}{\mathcal {W}}_k\bigg (\frac{1-(\varpi _{\alpha _{\sigma (k)}})^q}{(\varpi _{\alpha _{\sigma (k)}})^q}\bigg )^{\eta }\Bigg )^{\frac{1}{\eta }}}}} , \\ \root q \of {\frac{1}{1+\left( \sum _{k=1}^{u}{\mathcal {W}}_k\left( \frac{1-\beta _{\sigma (k)}^q}{\beta _{\sigma (k)}^q}\right) ^{\eta }\right) ^{\frac{1}{\eta }}}} e^{i2\pi \root q \of {\frac{1}{1+\Bigg (\sum _{k=1}^{u}{\mathcal {W}}_k\bigg (\frac{1-(\varpi _{\beta _{\sigma (k)}})^q}{(\varpi _{\beta _{\sigma (k)}})^q}\bigg )^{\eta }\Bigg )^{\frac{1}{\eta }}}}}, \\ \root q \of {1-\frac{1}{1+\left( \sum _{k=1}^{u}{\mathcal {W}}_k\left( \frac{\gamma _{\sigma (k)}^q}{1-\gamma _{\sigma (k)}^q}\right) ^{\eta }\right) ^{\frac{1}{\eta }}}} e^{i2\pi \root q \of {1-\frac{1}{1+\Bigg (\sum _{k=1}^{u}{\mathcal {W}}_k\bigg (\frac{(\varpi _{\gamma _{\sigma (k)}})^q}{1-\left( \varpi _{\gamma _{\sigma (k)}}\right) ^q}\bigg )^{\eta }\Bigg )^{\frac{1}{\eta }}}}} \end{array}\right) \end{aligned}$$

$$\begin{aligned} =\left( 0.761e^{i2\pi 0.773},0.489e^{i2\pi 0.508},0.450e^{i2\pi 0.468}\right) . \end{aligned}$$

#### Theorem 14

(Idempotency) Let $\digamma _k=\Bigg (\alpha _ke^{i2\pi \varpi _{\alpha _k}},\beta _ke^{i2\pi \varpi _{\beta _k}},\gamma _ke^{i2\pi \varpi _{\gamma _k}}\Bigg )$ $(k=1,2,\ldots ,u)$ be a set of CTSFNs such that $\digamma _k=\digamma$. Then

$$\begin{aligned} \mathrm{CTSDFOWGA}\left( \digamma _1,\digamma _2,\ldots ,\digamma _u \right) =\digamma . \end{aligned}$$

#### Theorem 15

(Monotonicity) Let $\digamma _k=\Bigg (\alpha _ke^{i2\pi \varpi _{\alpha _k}},\beta _ke^{i2\pi \varpi _{\beta _k}},\gamma _ke^{i2\pi \varpi _{\gamma _k}}\Bigg )$ and $\digamma _k'=\Bigg (\alpha _k'e^{i2\pi \varpi _{\alpha '_k}}, \beta _k'e^{i2\pi \varpi _{\beta '_k}},\gamma '_ke^{i2\pi \varpi _{\gamma '_k}}\Bigg )$ $(k=1,2,\ldots ,u)$ be two sets of CTSFNs. If $\alpha _k\le \alpha '_k, \beta _k\ge \beta '_k, \gamma _k\ge \gamma '_k$, $\varpi _{\alpha _k}\le \varpi _{\alpha '_k}, \varpi _{\beta _k}\ge \varpi _{\beta '_k},$ and $\varpi _{\gamma _k}\ge \varpi _{\gamma '_k}$ for all *k*. Then

$$\begin{aligned}&\mathrm{CTSDFOWGA}\left( \digamma _1,\digamma _2,\ldots ,\digamma _u\right) \\&\quad \preceq CTSDFOWGA\left( \digamma '_1,\digamma '_2,\ldots ,\digamma '_u\right) . \end{aligned}$$

#### Theorem 16

(Boundedness) Let $\digamma _k=\Big (\alpha _ke^{i2\pi \varpi _{\alpha _k}},\beta _ke^{i2\pi \varpi _{\beta _k}}, \gamma _ke^{i2\pi \varpi _{\gamma _k}}\Big ),$ $(k=1,2,3,\ldots ,u)$ a set of CTSFNs with $\digamma _\mathrm{min}=\mathrm{min}\left( \digamma _1,\digamma _2,\digamma _3,\ldots \ldots ,\digamma _u\right)$ and $\digamma _\mathrm{max}=\mathrm{max}\left( \digamma _1,\digamma _2,\digamma _3,\ldots ,\digamma _u\right)$. Then

$$\begin{aligned} \digamma _\mathrm{min} \preceq \mathrm{CTSDFOWGA}\left( \digamma _1,\digamma _2,\digamma _3,\ldots ,\digamma _u\right) \preceq \digamma _\mathrm{max}. \end{aligned}$$

## MCDM method under CTSF environment

In this section, we present an MCDM method under *CTSF* environment.

Let $\kappa =\{\kappa _1,\kappa _2,\ldots ,\kappa _l\}$ be set of alternatives, $\epsilon =\{\epsilon _1,\epsilon _2,\ldots ,\epsilon _s\}$ be a set of criteria. Let us consider ${\mathcal {W}}=\left( {\mathcal {W}}_1,{\mathcal {W}}_2,\ldots ,{\mathcal {W}}_s\right)$ such that ${\mathcal {W}}_j\in (0,1]$ and $\sum _{j=1}^{s}{\mathcal {W}}_j=1$ as the weight vector of the criteria which is determined by decision-makers. The steps of the MCDM method are given as follows:

***Step 1:*** The evaluation of the alternative $\kappa _i$ according to criteria $\epsilon _j$ performed by decision-maker. It can be written as $\zeta _{yj} (j=1,2,\ldots ,s; y=1,2,\ldots ,t)$. Hence, CTSF-decision matrix $\mathcal {DM}=[\zeta _{yj}]_{t\times s}$ can be constructed as follows:

$$\begin{aligned} \mathcal {DM}=[\zeta _{yj}]_{t\times s}=\left( \begin{array}{c@{\quad }c@{\quad }c@{\quad }c} \zeta _{11} &{} \zeta _{12} &{} \cdots &{} \zeta _{1s} \\ \zeta _{21} &{} \zeta _{22} &{} \cdots &{} \zeta _{2s} \\ \vdots &{} \vdots &{} \cdots &{} \vdots \\ \zeta _{t1} &{} \zeta _{t2} &{} \cdots &{} \zeta _{ts} \\ \end{array} \right) . \end{aligned}$$

Here $$\zeta _{yj}=\Big (\alpha _{yj} e^{i2\pi \varpi _{yj}},\beta _{yj} e^{i2\pi \varpi _{yj}},\gamma _{yj} e^{i2\pi \varpi _{yj}}\Big )$$.

***Step 2:*** Find the aggregated value denoted by ${\mathbb {A}}_y$ $(y=1,2,3,\ldots ,l)$ using the *CTSDFWAA* operators.

***Step 3:*** Find the score values, for each ${\mathbb {A}}_y y=1,2,3,\ldots ,l$

***Step 4:*** Choose the alternative which has a maximum score value.

### Application

The COVID-19 outbreak first appeared in Wuhan city of China in December 2019 and spread rapidly all over the world [70, 71]. Until May 5, 2021, 153,790,183 people were infected with the COVID-19 and 3,218,080 people died [72]. Also, the spread of COVID-19 still continuous. In some papers, mathematical analysis revealing the spread of such a deathly disease have been presented [73–75].

In this section, an application of the proposed method to determine a patient infected by COVID-19 is presented. In this application, after we discuss by infectious diseases physician, we determine the criteria as a basic symptoms of COVID-19. Set of the symptoms is considered as $\epsilon =\{\epsilon _1=\text { Fever } ,\epsilon _2=\text { headache }, \epsilon _3=\text { dyspnea }, \epsilon _4=\text { cough }\}$. Also, we consider the five patients $p_1,p_2,p_3,p_4$ and $p_5$. For each of patients, data measured along with 14 days according to symptoms are given in Tables 3, 4, 5 and 6.

**Table 3:** Fever values of patients measured for 14 days

	1	2	3	4	5	6	7	8	9
$p_1$	37.80	38.00	38.20	38.60	39.00	39.30	39.50	39.00	38.60
$p_2$	38.10	38.50	38.80	39.10	39.40	40.00	40.10	39.50	39.20
$p_3$	37.50	38.10	38.60	38.20	38.10	38.00	37.70	37.50	37.20
$p_4$	38.20	38.80	39.00	39.60	39.60	40.00	40.20	39.50	39.00
$p_5$	36.80	36.60	37.30	38.00	38.30	38.80	38.10	37.70	37.20

10	11	12	13	14					
38.10	37.80	37.50	37.20	37.00					
38.70	38.30	38.00	37.70	37.40					
37.00	37.40	37.10	36.80	36.60					
38.60	38.10	37.70	37.40	37.00					
37.20	36.80	36.60	36.90	36.50

**Table 4:** Headache values of patients measured for 14 days

	1	2	3	4	5	6	7	8	9	10	11	12	13	14
$p_1$	3.7	5.5	5.8	6.2	6.8	7.7	8.2	9.3	9.4	9.5	8.6	7.4	5.6	3.4
$p_2$	9.3	9.2	9.4	8.8	8.7	8.6	9.6	6.8	6.8	6.6	5.6	4.7	3.9	3.6
$p_3$	1.5	2.4	3.2	3.8	6.6	5.8	6.8	6.5	6.9	8.3	9.4	9.6	9.8	8.7
$p_4$	6.7	6.8	6.3	6.6	9.6	9.7	9.5	9.4	9.7	8.9	9.1	6.2	4.3	3.8
$p_5$	3.6	3.7	3.9	5.2	5.8	6.8	6.9	6.9	7.2	8.1	6.9	6.5	5.9	3.8

**Table 5:** Dyspnea values of patients measured for 14 days

	1	2	3	4	5	6	7	8	9	10	11	12	13	14
$p_1$	2.8	3.3	3.9	4.8	5.9	6.4	6.9	7.9	9.6	9.9	9.7	9.6	6.9	6.4
$p_2$	9.8	9.9	9.5	9.3	8.4	6.9	6.5	6.2	5.6	3.9	3.8	3.5	3.4	3.6
$p_3$	1.9	2.8	3.3	3.8	3.9	6.8	7.9	8.8	9.3	6.9	6.5	5.8	5.3	4.9
$p_4$	9.7	9.2	9.6	6.9	6.5	3.9	6.1	6.9	6.9	9.8	9.9	9.9	9.8	9.9
$p_5$	2.2	2.5	2.7	3.1	3.9	3.8	6.7	5.4	6.9	6.6	6.9	8.9	9.8	9.8

**Table 6:** Cough values of patients measured for 14 days

	1	2	3	4	5	6	7	8	9	10	11	12	13	14
$p_1$	3.1	3.4	4.8	6.7	8.6	8.2	8.3	6.6	6.2	5.4	3.9	3.5	3.6	3.4
$p_2$	3.9	6.1	6.3	6.8	6.9	8.8	9.7	9.8	9.9	9.8	9.8	9.9	6.9	6.8
$p_3$	2.9	3.6	3.9	5.9	6.1	6.8	6.9	6.9	9.3	9.9	9.7	9.8	9.9	9.8
$p_4$	9.7	9.9	9.6	9.9	9.8	6.9	6.5	6.9	6.9	3.9	3.2	3.1	2.8	2.7
$p_5$	3.8	3.9	5.5	6.7	6.8	6.9	6.7	8.8	7.8	7.9	8.9	9.3	9.8	9.8

Here we transform data given in tables to CTSFN. We will only explain transforming process of data given in Table 3. Other transforming of the tables will not be showed.

We establish amplitude terms and phase terms according to fever degree and days, respectively. First, we classify the degree of fever for MD (38.6-39.5 ), NeD(37.6-38.5 ), and NMD (36.5-37.5). Then we assign values from 0.1 to 0.9 for fever intervals. This is shown in Table 7.

**Table 7:** Classification of measured fever degrees according to MD, NeD, and NMD

	0.1	0.2	0.3	0.4	0.5	0.6	0.7	0.8	0.9
MD	38.6	38.7	38.8	38.9	39	39.1	39.2	39.3	39.4
NeD	37.7	37.8	37.9	38	38.1	38.2	38.3	38.4	38.5
NMD	36.7	36.8	36.9	37	37.1	37.2	37.3	37.4	37.5

For each of MD, NeD and NMD, we find the arithmetic mean of the fever values. Then we assign a value from 0.1 to 0.9.

and

We divide the number of MD, NeD and NMD days by 14 for each patient to obtain the phase terms. For example, according to Table 3, we see that fever of $p_1$ is in MD for 6 days, in NeD for 5 days and in NMD for 3 days. Then phase terms for MD, NeD and NMD are $\frac{6}{14}=0.43, \frac{5}{14}=0.36,$ and $\frac{3}{14}=0.21$. All of phase terms for patients are shown in Table .

**Table 8:** Arithmetic mean (AM) of fever values for MD, NeD, and NMD

	MD	NeD	NMD
$p_1$	39.00	37.98	37.23
$p_2$	39.35	38.12	37.40
$p_3$	38.60	38.02	37.14
$p_4$	39.37	38.00	37.20
$p_5$	38.80	38.03	36.88

**Table 9:** MD, NeD, and NMD values in [0,1] for patients

	MD	NeD	NMD
$p_1$	0.5	0.4	0.6
$p_2$	0.9	0.5	0.8
$p_3$	0.1	0.4	0.5
$p_4$	0.9	0.4	0.6
$p_5$	0.3	0.4	0.3

**Table 10:** Phase terms

	MD	NeD	NMD
$p_1$	0.43	0.36	0.21
$p_2$	0.57	0.36	0.07
$p_3$	0.07	0.36	0.57
$p_4$	0.64	0.21	0.14
$p_5$	0.07	0.29	0.64

In a similar way, we can obtain all of the CTSF values for each of the patients with respect to symptoms using following classification tables for other symptoms.

**Table 11:** Classification of measured headache degrees according to MD, NeD, and NMD

	0.1	0.2	0.3	0.4	0.5	0.6	0.7	0.8	0.9
MD	7.3	7.6	7.9	8.2	8.5	8.8	9.1	9.4	9.7
NeD	4.3	4.6	4.9	5.2	5.5	5.8	6.1	6.4	6.7
NMD	1.3	1.6	1.9	2.2	2.5	2.8	3.1	3.4	3.7

Here we use the steps of the proposed method.

***Step 1:*** $\mathcal {DM}$ matrix is constructed by taking into consideration the above data.

$$\begin{aligned} \mathcal {DM}=\left[ \begin{array}{cc} (0.5e^{i2\pi 0.43}, 0.4e^{i2\pi 0.36}, 0.6e^{i2\pi 0.21}) &{} (0.5e^{i2\pi 0.50}, 0.7e^{i2\pi 0.36},0.8e^{i2\pi 0.14}) \\ ( 0.9e^{i2\pi 0.57}, 0.5e^{i2\pi 0.36}, 0.8e^{i2\pi 0.07}) &{} (0.7e^{i2\pi 0.50}, 0.7e^{i2\pi 0.36}, 0.9e^{i2\pi 0.14}) \\ (0.1e^{i2\pi 0.07}, 0.4e^{i2\pi 0.36}, 0.5e^{i2\pi 0.57}) &{} (0.7e^{i2\pi 0.36}, 0.8e^{i2\pi 0.36}, 0.5e^{i2\pi 0.29}) \\ (0.9e^{i2\pi 0.64},0.8e^{i2\pi 0.36}, 0.6e^{i2\pi 0.14}) &{} (0.8e^{i2\pi 0.50}, 0.7e^{i2\pi 0.43}, 0.9e^{i2\pi 0.07}) \\ (0.3e^{i2\pi 0.07}, 0.4e^{i2\pi 0.29}, 0.3e^{i2\pi 0.64}) &{} (0.2e^{i2\pi 0.14}, 0.8e^{i2\pi 0.57},0.9e^{i2\pi 0.29}) \\ \end{array} \right. \\ \left. \begin{array}{cc} (0.8e^{i2\pi 0.36},0.7e^{i2\pi 0.43},0.8e^{i2\pi 0.21}) &{} (0.5e^{i2\pi 0.21},0.6e^{i2\pi 0.36},0.8e^{i2\pi 0.43}) \\ (0.8e^{i2\pi 0.36},0.8e^{i2\pi 0.29}, 0.9e^{i2\pi 0.36}) &{} (0.8e^{i2\pi 0.50},0.9e^{i2\pi 0.43},0.9e^{i2\pi 0.07}) \\ (0.5e^{i2\pi 0.21},0.7e^{i2\pi 0.43},0.7e^{i2\pi 0.36}) &{} (0.9e^{i2\pi 0.43},0.8e^{i2\pi 0.36},0.8e^{i2\pi 0.21}) \\ (0.9e^{i2\pi 0.57},0.8e^{i2\pi 0.36}, 0.9e^{i2\pi 0.07}) &{} (0.9e^{i2\pi 0.36},0.9e^{i2\pi 0.29},0.7e^{i2\pi 0.36}) \\ (0.8e^{i2\pi 0.21},0.8e^{i2\pi 0.36}, 0.7e^{i2\pi 0.43}) &{} (0.6e^{i2\pi 0.50},0.8e^{i2\pi 0.36},0.9e^{i2\pi 0.14}) \\ \end{array} \right] \end{aligned}$$

Here we consider $q=9$.

**Step 2**: Aggregated values are found for each of the patients by applying *CTSDFWAA* and *CTSDFWGA* operators to rows of the $\mathcal {MD}$.

**Table 12:** Obtaining results for headache using Tables 4 and 11

	MD	NeD	NMD			MD	NeD	NMD			MD	NeD	NMD
$p_1$	8.59	5.98	3.55		$p_1$	0.5	0.7	0.8		$p_1$	0.50	0.36	0.14
$p_2$	9.09	6.10	3.75		$p_2$	0.7	0.7	0.9		$p_2$	0.50	0.36	0.14
$p_3$	9.16	6.52	2.73		$p_3$	0.7	0.8	0.5		$p_3$	0.36	0.36	0.29
$p_4$	9.41	6.15	3.80		$p_4$	0.8	0.7	0.9		$p_4$	0.50	0.43	0.07
$p_5$	7.65	6.36	3.75		$p_5$	0.2	0.8	0.9		$p_5$	0.14	0.57	0.29
AM of headache degrees						MD, NeD, and NMD values				Phase terms

**Table 13:** Classification of measured dyspnea degrees according to MD, NeD, and NMD

	0.1	0.2	0.3	0.4	0.5	0.6	0.7	0.8	0.9
MD	7.3	7.6	7.9	8.2	8.5	8.8	9.1	9.4	9.7
NeD	4.3	4.6	4.9	5.2	5.5	5.8	6.1	6.4	6.7
NMD	1.3	1.6	1.9	2.2	2.5	2.8	3.1	3.4	3.7

**Table 14:** Obtaining results for dyspnea using Tables 5 and 13

	MD	NeD	NMD			MD	NeD	NMD			MD	NeD	NMD
$p_1$	9.34	6.22	3.33		$p_1$	0.8	0.7	0.8		$p_1$	0.36	0.43	0.21
$p_2$	9.38	6.30	3.64		$p_2$	0.8	0.8	0.9		$p_2$	0.36	0.29	0.36
$p_3$	8.67	6.03	3.14		$p_3$	0.5	0.7	0.7		$p_3$	0.21	0.43	0.36
$p_4$	9.73	6.66	3.90		$p_4$	0.9	0.9	0.9		$p_4$	0.57	0.36	0.07
$p_5$	9.50	6.50	3.03		$p_5$	0.8	0.8	0.7		$p_5$	0.21	0.36	0.43
AM of dyspnea degrees						MD, NeD, and NMD values				Phase terms

**Table 15:** Classification of measured cough degrees according to MD, NeD, and NMD

	0.1	0.2	0.3	0.4	0.5	0.6	0.7	0.8	0.9
MD	7.3	7.6	7.9	8.2	8.5	8.8	9.1	9.4	9.7
NeD	4.3	4.6	4.9	5.2	5.5	5.8	6.1	6.4	6.7
NMD	1.3	1.6	1.9	2.2	2.5	2.8	3.1	3.4	3.7

**Table 16:** Obtaining results for cough using Tables 6 and 15

	MD	NeD	NMD			MD	NeD	NMD			MD	NeD	NMD
$p_1$	8.37	5.94	3.48		$p_1$	0.5	0.6	0.8		$p_1$	0.21	0.36	0.43
$p_2$	9.67	6.63	3.90		$p_2$	0.8	0.9	0.9		$p_2$	0.50	0.43	0.07
$p_3$	9.73	6.52	3.47		$p_3$	0.9	0.8	0.8		$p_3$	0.43	0.36	0.21
$p_4$	9.78	6.80	3.14		$p_4$	0.9	0.9	0.7		$p_4$	0.36	0.29	0.36
$p_5$	8.90	6.52	3.85		$p_5$	0.6	0.8	0.9		$p_5$	0.50	0.36	0.14
AM of cough degree						MD, NeD, and NMD values				Phase terms

**Table 17:** CTSFSs obtained using CTSDFWAA and CTSDFWGA

	*CTSDFWAA*	*CTSDFWGA*
$p_{1}$	$(0.709e^{i2\pi 0.429},0.448e^{i2\pi 0.368}, 0.655e^{i2\pi 0.174})$	$( 0.520e^{i2\pi 0.256}, 0.448e^{i2\pi 0.368}, 0.769e^{i2\pi 0.359})$
$p_{2}$	$(0.752e^{i2\pi 0.524},0.559e^{i2\pi 0.319},0.851e^{i2\pi 0.076})$	$( 0.790e^{i2\pi 0.405}, 0.559e^{i2\pi 0.319}, 0.880e^{i2\pi 0.312})$
$p_{3}$	$(0.788e^{i2\pi 0.364},0.449e^{i2\pi 0.368},0.538e^{i2\pi 0.254})$	$(0.112e^{i2\pi 0.080},0.449e^{i2\pi 0.368},0.704e^{i2\pi 0.509})$
$p_{4}$	$(0.875e^{i2\pi 0.424},0.449e^{i2\pi 0.239},0.849e^{i2\pi 0.299})$	$( 0.875e^{i2\pi 0.424},0.449e^{i2\pi 0.239},0.849e^{i2\pi 0.299})$
$p_{5}$	$(0.711e^{i2\pi 0.418},0.449e^{i2\pi 0.315},0.337e^{i2\pi 0.171})$	$(0.245e^{i2\pi 0.080}, 0.449e^{i2\pi 0.315}, 0.831e^{i2\pi 0.574})$

**Table 18:** Score values for CTSDFWAA values

	$\eta =1$	$\eta =2$	$\eta =3$	$\eta =4$	$\eta =5$	$\eta =6$	$\eta =7$	$\eta =8$	$\eta =9$	$\eta =10$
$P_{1}$	0.505	0.515	0.510	0.523	0.524	0.525	0.526	0.527	0.527	0.528
$P_{2}$	0.460	0.475	0.483	0.488	0.490	0.492	0.493	0.494	0.495	0.496
$P_{3}$	0.528	0.554	0.567	0.574	0.578	0.581	0.583	0.585	0.586	0.587
$P_{4}$	**0.559**	**0.576**	**0.583**	**0.587**	**0.589**	**0.591**	**0.592**	**0.593**	**0.594**	**0.594**
$P_{5}$	0.511	0.520	0.524	0.526	0.527	0.528	0.529	0.530	0.530	0.531

**Table 19:** Score values for CTSDFWGA values

	$\eta =1$	$\eta =2$	$\eta =3$	$\eta =4$	$\eta =5$	$\eta =6$	$\eta =7$	$\eta =8$	$\eta =9$	$\eta =10$
$P_{1}$	0.477	0.473	0.471	0.470	0.469	0.469	0.469	0.468	0.468	0.468
$P_{2}$	0.449	0.436	0.429	0.425	0.422	0.420	0.419	0.418	0.418	0.417
$P_{3}$	0.489	0.482	**0.478**	**0.475**	**0.473**	**0.472**	** 0.471**	**0.470**	**0.470**	**0.469**
$P_{4}$	**0.518**	**0.490**	0.474	0.464	0.459	0.455	0.452	0.450	0.448	0.447
$P_{5}$	0.451	0.429	0.420	0.414	0.411	0.409	0.408	0.407	0.406	0.405

***Step 3***: Using Eq. 1, score values of the aggregated values are obtained as follows:

**Table:** 

	SVs according to CTSDFWAA op.	SVs according to CTSDFWGA op.
$p_{1}$	0.505	0.447
$p_{2}$	0.460	0.449
$p_{3}$	0.528	0.489
$p_{4}$	**0.559**	**0.518**
$p_{5}$	0.511	0.451

**Step 4**: According to Table 4.1, $p_4$ is suffer from COVID-19.

## Sensitivity analyses and discussion

In this section, we compute the score values of the patients according to CTSDFWAA and CTSDFWGA values for “$\eta =1,2,\ldots ,10$”.

According to Tables 18 and 19, we see that for $\eta =1,2,\ldots ,10$, results obtained using CTSDFWAA operator match by medical results. By results, $P_4$ was infected by COVID-19. For $\eta =1,2$ results from both operators are consistent. For $\eta =3,4,\ldots ,10$ according to results obtained using CTSDFWGA operator $P_3$ was infected by COVID-19. Also, this matches the medical results made by a medical doctor. In this study, we consider only four symptoms. However, the epidemic of the COVID-19 continue all over the worlds and medical researchers encounter some new symptoms of COVID-19. Here we give a simple example to show the trueness of the proposed method, this is a restriction of our study. We think that this study may be a reference point for researchers who want to study clustering and medical diagnosis with large data.

## Conclusion

In this paper, weakness of score and accuracy function defined by Ali et al. [57] was pointed out and new score and accuracy functions were defined for CTSFNs. Set theoretical operations was introduced and some aggregation operators based on Dombi t-norms and t-conorms were defined under CTSF environment with their examples. Also, some properties of the proposed aggregation operators were investigated. Furthermore, an MCDM method was developed based on proposed aggregation operators and score function. Moreover, an application of the developed method, including determining the COVID-19, was presented by transforming the real data to CTSF data. We see that obtained results match real results. We also pointed out some restrictions and their reasons. In future, our targets are to study other aggregation operators such as Hamacher and Bonferroni, similarity measures, distance measures and decision-making methods based on TOPSIS, VIKOR, AHP, etc. By transferring the algorithm of the proposed method to the computer program, our analysis for a limited number of patients can be made under big data and by considering more parameters. We hope that this study will provide a useful perspective for researchers working on decision-making.
